# Spatiotemporal invasion dynamics of SARS-CoV-2 lineage B.1.1.7 emergence

**DOI:** 10.1126/science.abj0113

**Published:** 2021-08-20

**Authors:** Moritz U. G. Kraemer, Verity Hill, Christopher Ruis, Simon Dellicour, Sumali Bajaj, John T. McCrone, Guy Baele, Kris V. Parag, Anya Lindström Battle, Bernardo Gutierrez, Ben Jackson, Rachel Colquhoun, Áine O’Toole, Brennan Klein, Alessandro Vespignani, COVID-19 Genomics UK (COG-UK) Consortium, Erik Volz, Nuno R. Faria, David M. Aanensen, Nicholas J. Loman, Louis du Plessis, Simon Cauchemez, Andrew Rambaut, Samuel V. Scarpino, Oliver G. Pybus

**Affiliations:** 1 Department of Zoology, University of Oxford, Oxford, UK.; 2 Institute of Evolutionary Biology, University of Edinburgh, Edinburgh, UK.; 3 Molecular Immunity Unit, Department of Medicine, Cambridge University, Cambridge, UK.; 4 Spatial Epidemiology Lab (SpELL), Université Libre de Bruxelles, Bruxelles, Belgium.; 5 Department of Microbiology, Immunology and Transplantation, Rega Institute, KU Leuven, 3000 Leuven, Belgium.; 6 MRC Centre for Global Infectious Disease Analysis, Jameel Institute for Disease and Emergency Analytics, Imperial College London, London, UK.; 7 Department of Plant Sciences, University of Oxford, Oxford, UK.; 8 Network Science Institute, Northeastern University, Boston, USA.; 9 Instituto de Medicina Tropical, Faculdade de Medicina da Universidade de Sao Paulo, Sao Paulo, Brazil.; 10 Centre for Genomic Pathogen Surveillance, Wellcome Genome Campus, Hinxton, UK.; 11 Big Data Institute, Li Ka Shing Centre for Health Information and Discovery, Nuffield Department of Medicine, University of Oxford, Oxford, UK.; 12 Institute of Microbiology and Infection, University of Birmingham, Birmingham, UK.; 13 Mathematical Modelling of Infectious Diseases Unit, Institut Pasteur, UMR2000, CNRS, Paris, France.; 14 Vermont Complex Systems Center, University of Vermont, Burlington, USA.; 15 Santa Fe Institute, Santa Fe, USA.; 16 Department of Pathobiology and Population Sciences, Royal Veterinary College London, London, UK.

## Abstract

Understanding the causes and consequences of the emergence of severe acute respiratory syndrome coronavirus 2 (SARS-CoV-2) variants of concern is crucial to pandemic control yet difficult to achieve because they arise in the context of variable human behavior and immunity. We investigated the spatial invasion dynamics of lineage B.1.1.7 by jointly analyzing UK human mobility, virus genomes, and community-based polymerase chain reaction data. We identified a multistage spatial invasion process in which early B.1.1.7 growth rates were associated with mobility and asymmetric lineage export from a dominant source location, enhancing the effects of B.1.1.7’s increased intrinsic transmissibility. We further explored how B.1.1.7 spread was shaped by nonpharmaceutical interventions and spatial variation in previous attack rates. Our findings show that careful accounting of the behavioral and epidemiological context within which variants of concern emerge is necessary to interpret correctly their observed relative growth rates.

## Fueling outbreaks

The B.1.1.7 lineage of severe acute respiratory syndrome coronavirus 2 (SARS-CoV-2) has caused fast-spreading outbreaks globally. Intrinsically, this variant has greater transmissibility than its predecessors, but this capacity has been amplified in some circumstances to tragic effect by a combination of human behavior and local immunity. What are the extrinsic factors that help or hinder the rapid dissemination of variants? Kraemer *et al*. explored the invasion dynamics of B.1.1.7. in fine detail, from its location of origin in Kent, UK, to its heterogenous spread around the country. A combination of mobile phone and virus data including more than 17,000 genomes shows how distinct phases of dispersal were related to intensity of mobility and the timing of lockdowns. As the local outbreaks grew, importation from the London source area became less important. Had B.1.1.7. emerged at a slightly different time of year, its impact might have been different. —CA

## Teaser

Disentangling the factors that contribute to the rapid spread of virus variants is essential for understanding their epidemiological consequences.

The severe acute respiratory syndrome coronavirus 2 (SARS-CoV-2) lineage B.1.1.7 expanded rapidly across the United Kingdom (*1*, *2*) in late 2020 and subsequently spread internationally (*3*, *4*). As of 19 January 2021 (date of the most recent sample in our dataset), B.1.1.7 had reached all but five counties of Wales, Scotland, Northern Ireland, and England, with onward transmission in each. Restrictions on international travel were enacted to contain B.1.1.7’s spread; however, genomic surveillance has since detected the presence and growth of the lineage in many countries worldwide (*4*, *5*). Analyses of genomic, laboratory, secondary contact, and aggregated epidemiological data estimate higher transmissibility of B.1.1.7 compared with previous SARS-CoV-2 lineages (*1*, *6*–*9*) and potentially a greater risk of hospitalization (*10*–*13*). The spatial heterogeneity of SARS-CoV-2 transmission—and of emerging infectious diseases in general—can have profound effects on the local likelihood and intensity of transmission, final epidemic size, and immunity (*14*–*22*). More specifically, estimates of B.1.1.7’s increased relative transmissibility declined during its emergence in the UK (*7*, *9*); understanding why this occurred is necessary if we are to respond effectively to future SARS-CoV-2 variants. We reconstructed and quantified the spatial dynamics of B.1.1.7’s emergence and investigated how human mobility and heterogeneity in previous exposure contributed to B.1.1.7’s initial spread and evaluation of higher transmissibility.

## Spatial expansion and source sink dynamics of B.1.1.7 in the UK

**Fig. 1.**
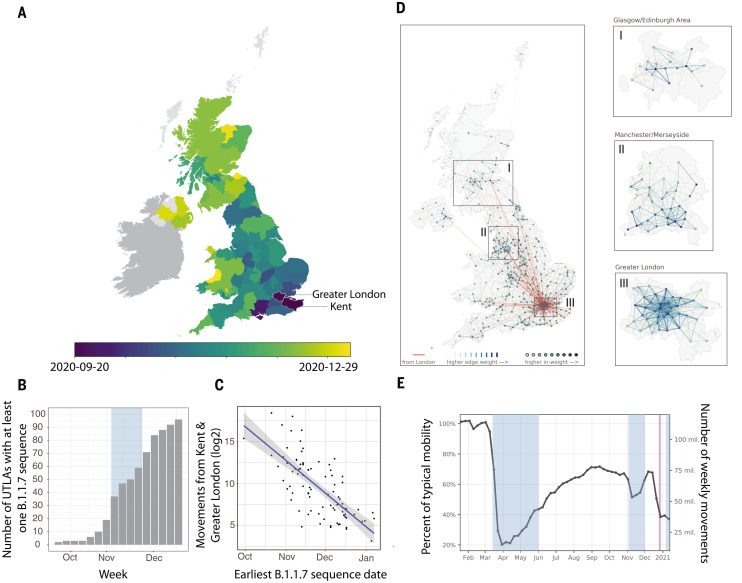
Human mobility and spatial expansion of B.1.1.7 across the UK. (**A**) Map at the UTLA level of arrival dates of lineage B.1.1.7. Darker colors indicate earlier dates, and lighter colors indicate later dates. Arrival time is defined as the earliest sampling date of a B.1.1.7 genomic sequence in each UTLA. (**B**) Cumulative number of UTLAs in which B.1.1.7 has been detected, in 7-day intervals. The blue shaded area indicates the period of the second lockdown in England. (**C**) Relationship between the arrival time of B.1.1.7 and estimated number of movements from Kent and London during February 2020 for each UTLA (Pearson’s *r* = –0.73; 95% CI: –0.61, –0.81; *P* < 0.001) (materials and methods). (**D**) Human mobility at the UK local authority district level (LAD) (table S2) during the epidemiological week 29 November to 5 December 2020. Thicker lines (edges) indicate more movements between regions. Nodes with larger absolute incoming movements are indicated with darker colors. Red lines indicate movements from Greater London. (Insets I, II, and III) Mobility within three UK metropolitan areas. (**E**) Trends in human mobility across the UK (indicating movements between but not within LADs). The blue shaded areas indicate the period of the first, second, and third lockdown in England. Dark red indicates the timing (20 December 2020) of the Tier 4 restrictions imposed in southeast England, including London (*56*).

B.1.1.7 can be first detected in COVID-19 Genomics UK Consortium (COG-UK) genome data in Kent on 20 September 2020 and spread quickly across the UK, with each week adding detections in approximately seven new upper-tier local authorities (UTLAs) (Fig. 1, A and B, and table S2). B.1.1.7 was already reported in several UTLAs before the start of the second English lockdown (5 November 2020). By the end of that lockdown (2 December 2020), B.1.1.7 was widespread throughout the UK (Fig. 1, A and B).

The spatial expansion of SARS-CoV-2 lineages [for example, (*16*, *23*)] can be tracked by using data from the UK’s national surveillance of SARS-CoV-2 genomes (*24*). By combining these data with aggregated mobile phone data, we examined the dissemination of B.1.1.7 through human mobility, from its likely location of emergence (Kent and Greater London) to other UK regions (Fig. 1, D and E, and supplementary materials, materials and methods). Human mobility among UK regions increased at the end of the second English lockdown, from 55 million to 75 million weekly movements (Fig. 1E). Because of its centrality, Greater London exhibits an important connective role in the UK human movement network (Fig. 1D; red lines indicate the week the second lockdown was eased). Compared with that of previous weeks, movements out of Greater London were more frequent and reached more destinations (fig. S1). For each UTLA, we found that the date of first detection of B.1.1.7 is predicted well by human mobility from Kent and Greater London to that UTLA [Pearson’s correlation coefficient (*r*) = –0.73; 95% confidence interval (CI): –0.61, –0.81; Akaike information criteria (AIC) = 734] (Fig. 1C) and similarly well by using movements from Kent and Greater London separately (fig. S2). This correlation strengthens through time as new locations of B.1.1.7 detection are added (fig. S3) and is robust to changes in human mobility through time in among-region human movement (Pearson’s *r* = –0.44; 95% CI: –0.16, –0.65; *P* < 0.01; mobility data through 23 January 2021) (materials and methods). Geographic distance from Greater London correlates less strongly with B.1.1.7 arrival times (Pearson’s *r* = 0.60; 95% CI: 0.44 to 0.71; AIC = 763) (fig. S4).

**Fig. 2.**
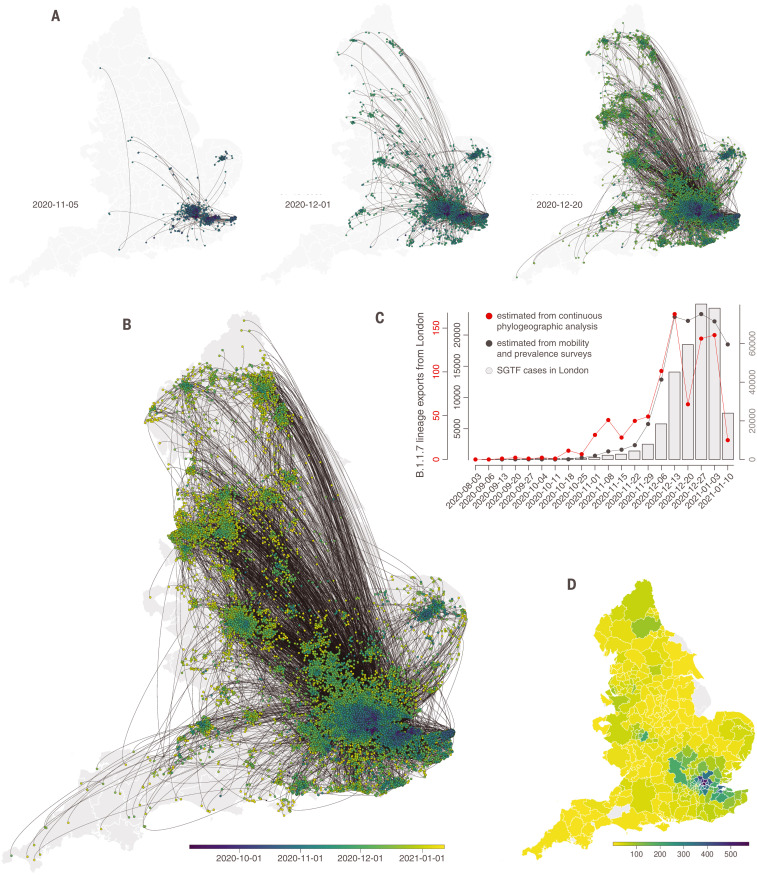
Spatial emergence dynamics of SARS-CoV-2 lineage B.1.1.7 in England. (**A** and **B**) Continuous phylogeographic reconstruction with phylogeny nodes colored according to their time of occurrence and dispersal direction of phylogeny branches indicated by edge curvature (counterclockwise). From left to right, data to 5 November, 1 December, and 20 December 2020, respectively. (B) Map of the entire reconstruction, up to 19 January 2021. (**C**) Estimated number of weekly exports of lineage B.1.1.7 from the Greater London area, inferred from the continuous phylogeographic analysis (red), and estimated from mobility and prevalence survey data (black). (**D**) Estimated number of cumulative B.1.1.7 introductions inferred from phylogeographic analysis into each administrative area (UTLA) by 12 December 2020.

To understand better the spatial dispersal of B.1.1.7 during its emergence, we reconstructed its spread across England using large-scale phylogeographic analysis (*25*–*27*). We analyzed 17,716 B.1.1.7 genomes collected between 20 September 2020 and 19 January 2021 (Fig. 2 and fig. S5), collated from polymerase chain reaction (PCR)–positive community samples that represent a random selection of SARS-CoV-2–positive samples (*28*). These genomes represent ~4% of UK B.1.1.7 cases during the study period [*n* = 460,510 estimated tests with PCR S-gene target failure (SGTF) between 20 September 2020 and 19 January 2021]. Samples per location (UTLA) and per week in the SGTF and whole-genome datasets are strongly correlated (Pearson’s *r* = 0.69; 95% CI: 0.63 – 0.73; *P* < 0.001) (fig. S6) (*7*), making it feasible to reconstruct B.1.1.7 expansion history by using phylogeographic approaches (*29*).

**Fig. 3.**
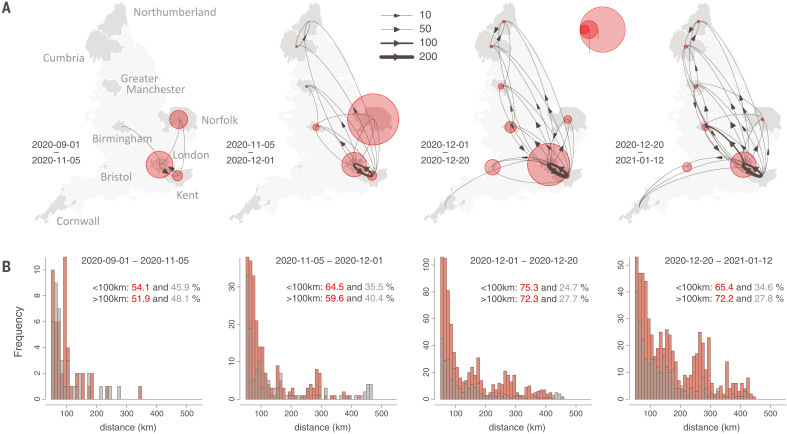
Spatial structure of B.1.1.7 lineage dispersal in England from phylogeographic reconstruction. (**A**) Curved arrows and line thicknesses indicate the direction and intensity of B.1.1.7 lineage flows among regions. Red circles indicate, for a given location, the ratio of inferred local movements to inferred importations into that location. Four time periods are shown (left to right) and roughly correspond to (i) before second lockdown, (ii) second lockdown, (iii) after second lockdown, and (iv) implementation of Tier 4 restrictions in southeast England. (**B**) Distribution of the geographic distances of phylogenetic lineage movement events (>50 km). Those from Greater London are in red, and those from other locations are in gray.

We identified distinct phases to the emergence of B.1.1.7. Initially, during the second English lockdown, most (71.2%) B.1.1.7 phylogenetic branch movements originated and ended in Greater London or Kent; long-distance dispersal events were relatively infrequent (Figs. 2 and 3). After the lockdown ended, and new cases in London subsequently rose rapidly, observed virus lineage movements from southeast England to other regions increased, and other large cities started to exhibit local transmission (Figs. 2 and 3). This phase of a growing number of exported B.1.1.7 cases from London and environs stabilized in mid-December and coincided with reduced mobility from Greater London (Tier 4 restrictions were announced on 20 December 2020 and entailed a “Stay at home” order, closure of nonessential shops and hospitality, and strict limitations on household mixing) (Figs. 1E and 2C). However, the total number of B.1.1.7 lineage exports did not immediately decline because the growing number of B.1.1.7 cases in southeast England offset the decline in outward travel (Fig. 2C) (*30*), indicating a limited effect of delayed action on B.1.1.7 spread from Greater London. Our analysis did not allow us to establish a causal link between nonpharmaceutical interventions (NPIs) and their impact on lineage exportations, so these results should be interpreted with caution.

By combining mobility and SGTF data with estimates of the proportion of the population testing SARS-CoV-2–positive (materials and methods), we can estimate the frequency of B.1.1.7 export from Greater London to other English regions (Fig. 2C and fig. S7) and explore its role in accelerating the lineage’s emergence. Using these combined data sources, we estimate that the number of B.1.1.7 case exports from Greater London rose during November (including during lockdown) from <600 to >12,000 in early December (Fig. 2C, gray curve), reflecting growth in B.1.1.7 infections in Greater London and an increase in human mobility among UK geographic regions across in late November (Fig. 1E). The estimated intensity of B.1.1.7 case exportation from Greater London remained high in December, peaking in mid-December at ~20,000 weekly exports, before declining in early January after the third national lockdown started on 5 January 2021. These estimates (Fig. 2C, gray curve) closely match the trends in lineage B.1.1.7 movement inferred from phylogeographic analysis (Fig. 2C, red curve), cross-validating both data sources (exports estimated by using each method are strongly correlated; Pearson’s *r* = 0.62; 95% CI: 0.61 to 0.64; *P* < 0.001) (fig. S8). Lineage exportation events estimated from genomic data are lower from late December onward, possibly owing to reporting lags in genomic data generation and/or delayed care-seeking because of the Christmas holidays (*31*). Our simple model assumes that nonsymptomatic infectious individuals are equally likely to travel (Fig. 2C, gray line), which may bias our estimates of infectious travellers upward.

B.1.1.7 dispersal dynamics shifted in late December to more bidirectional exchange of phylogenetic lineages in and out of Greater London (Fig. 3), coinciding with rapid growth in B.1.1.7 cases across England (*9*). Throughout, the weekly number of B.1.1.7 cases in a UTLA was positively associated with the number of B.1.1.7 lineage introductions into that UTLA during that week (Pearson’s *r* = 0.41, 0.76, 0.91, and 0.73, for October, November, December, and January, respectively; *P* < 0.001 for all; further analysis is provided in the supplementary materials) (fig. S6). We observed spatial heterogeneity in B.1.1.7 lineage importations; in the phylogeographic analysis, some locations received >500 inferred importations, despite our genomic dataset representing <4% of reported B.1.1.7 cases during the study period (Fig. 2D).

Detailed mapping of the spatial dynamics of SARS-CoV-2 lineages is difficult without comprehensive, well-sampled epidemiological and genomic data (*32*, *33*). However, the COG-UK data enables us to study dissemination trends by comparing inferred B.1.1.7 importations with within-location movements. Greater London (and to some extent Kent) acted as the main exporter of B.1.1.7 lineages to other UTLAs until mid-December 2020 (Fig. 3A). The longest (>100 km) and shortest (<100 km) dispersal events consistently originated from Greater London throughout the study period (Fig. 3B), primarily because of its large epidemic. However, the relative percentage of lineage movements that originated from Greater London approximately halved between September 2020 and January 2021 (table S1).

## Spatial heterogeneity in SARS-CoV-2 incidence and B.1.1.7 expansion

**Fig. 4.**
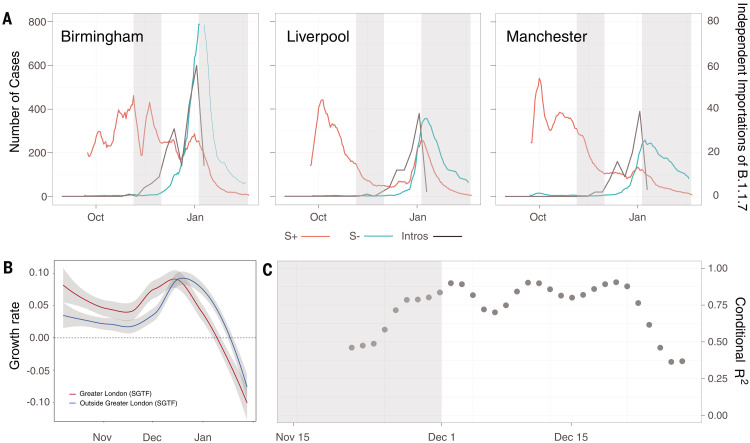
Case growth rates of B.1.1.7 are correlated with human mobility and attack rates across the UK. (**A**) Seven-day rolling average number of cases reported that had the SGTF (green) and cases reported with non-SGTF (red) for three selected LTLAs, Birmingham, Liverpool, and Manchester (table S2). The black line indicates the weekly number of independent introductions estimated from the phylogeographic analysis. The gray shaded area indicates the timing of the second (5 November to 1 December) and start of the third (5 January) English lockdown. (**B**) Rolling 7-day average growth rates of SGTF cases in Greater London (red line) and outside of Greater London (blue line). (**C**) Association between per-region (LTLA) difference between SGTF and non-SGTF case growth rates (corrected to account for differences through time in sampling intensity of SGTF cases) and number of B.1.1.7 importations into that region, as estimated from prevalence surveys and human mobility (gray dots) (Fig. 2C and materials and methods). The gray area shows the time of the second English lockdown. Modeling results for SGTF growth rates are shown in fig. S9, and regression results under different assumptions about the frequency of SGTF are shown in fig. S10.

Using SGTF PCR-positive tests as a proxy for B.1.1.7 infection (*34*), we next examined daily growth rates of SARS-CoV-2 cases at the UTLA level for SGTF and non-SGTF cases (excluding case data from 25 to 31 January to account for reporting and testing delays) (materials and methods) (*35*). Case growth rates immediately after the November 2020 lockdown were highest in regions of southeast England connected to Greater London and/or Kent (fig. S9). Acceleration in SGTF case growth rates in Greater London began in mid-November and preceded acceleration in other regions (Fig. 4B). At the UTLA level, growth rates of SGTF cases were higher than non-SGTF cases (fig. S9), a key observation used to support an increased transmissibility for B.1.1.7 (*7*, *9*).

We added to those findings by quantifying the import of B.1.1.7 cases from London and investigating the association of importation trends with lineage-specific case growth rates (materials and methods). Using our phylogeographic analysis results (Figs. 2 and 3), we found that growth in the rate of B.1.1.7 importation into a lower-tier local authority (LTLA) closely matches the early growth rate of SGTF cases in that LTLA (Birmingham, Liverpool, and Manchester are shown in Fig. 4A). We further calculated the per-region difference between SGTF and non-SGTF case growth rates [the estimated raw additive increase in SGTF growth rate is 0.0715, and the median multiplicative advantage is 1.576, assuming a generation time of 6.5 days, which is qualitatively similar to those reported previously (*7*, *9*), with the caveat that generation times may differ between B.1.1.7 and other lineages (*36*, *37*)] (fig. S11). The degree to which this difference is positively correlated with B.1.1.7 importation rate grew during the latter half of the November lockdown and remained very high [coefficient of determination (*R*^2^) > 0.75] until mid-December, before declining (the trend remains when accounting for uncertainty in the estimated number of infections across Greater London) (Fig. 4C and fig. S12). This result is robust to the data and methods used to estimate per-location B.1.1.7 importation rates (figs. S9 and S10). Accounting for continued export of B.1.1.7 from Greater London and Kent can explain in part why estimates of the growth advantage of B.1.1.7 declined during the second half of December 2020, before the implementation of tighter control measures (Tier 4, 20 December) (*7*, *9*).

## Human mobility and prior outbreaks as predictors of B.1.1.7 growth

**Fig. 5.**
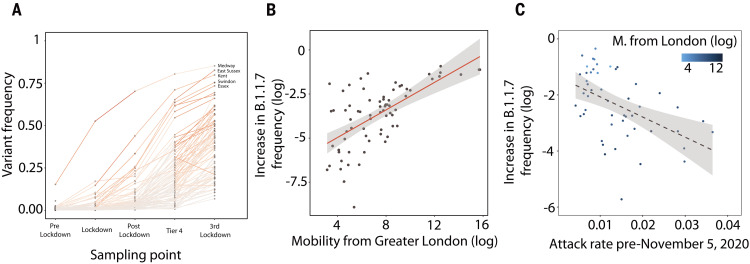
(**A**) Frequency of B.1.1.7 at the UTLA level at different sampling times. Pre-lockdown, dates before 5 November; lockdown, 5 to 30 November; post-lockdown, 1 to 15 December; Tier 4, 16 to 31 December; and the most recent sampling point, 1 to 12 January(materials and methods). (**B**) Increase in the frequency of B.1.1.7 sampled genomes between 2 and 16 December 2020 is associated with mobility from Greater London. (**C**) Increase in the frequency of B.1.1.7 sampled genomes at the UTLA level is associated with previous attack rates in each location. Results for equivalent analyses of SGTF data are similar and are provided in the supplementary materials.

The epicenter of SARS-CoV-2 transmission in the UK shifted during the November 2020 lockdown: between 1 September and 1 December 2020, ~80% of reported cases were reported outside London and southeast England, whereas those regions accounted for ~40% of all cases during 1 to 7 December. We sought to understand how, in each location, post-lockdown growth rates related to previous attack rates as well as travel inflow to that location. We investigated predictors of the increase in the relative frequency of B.1.1.7 genomes compared with that of other SARS-CoV-2 lineages (Fig. 5A) (*7*, *9*). In a multivariate model, we found that about half of the variation in the increase in B.1.1.7 relative frequency between 2 and 16 December is associated with human mobility from Greater London and attack rates before the November lockdown (Fig. 5, B and C). UTLAs with lower previous attack rates tended to have faster-increasing B.1.1.7 frequencies. We repeated this analysis using SGTF case frequency data and obtained similar results (*R*^2^ = 0.57, *P* < 0.001) (fig. S13). However, neither human mobility nor pre-lockdown attack rate were significant predictors of later changes. Instead, change in the relative frequency of B.1.1.7 genomes after 17 December was best predicted simply by its frequency on that date (*R*^2^ = 0.13, *P* < 0.01) (fig. S14), although a model identified through exhaustive search by using Bayesian information criteria (BIC) includes the “frequency of B.1.1.7 on 17 December,” an interaction between arrival time and “frequency of B.1.1.7 on 17 December,” and an interaction between incidence before the November lockdown and mobility from London (BIC 178.467; *R*^2^ = 0.68; *P* < 0.001) (fig. S14). Mobility from Greater London remains a significant predictor of B.1.1.7 growth after controlling for population size by means of both a multivariate regression and model-selection by using exhaustive search with both BIC and AIC.

## Conclusions, limitations, and future work

We found that the emergence of B.1.1.7 throughout the UK was associated with a high export frequency from a major source location that was identified only retrospectively. This pattern recapitulates at a national scale the role that international mobility played in the early spread of the SARS-CoV-2 pandemic (*38*–*40*). We conclude that the exceptionally rapid spatial spread and early growth rates of lineage B.1.1.7 likely reflect the combined effects of its higher intrinsic transmissibility (*1*, *7*, *9*) and the spatial structure of incidence and mobility before, during, and after the second lockdown in England (*41*).

Understanding what causes a new SARS-CoV-2 lineage to grow and replace preexisting lineages is a complex problem. In addition to virus genetic changes to relevant phenotypes (such as per-contact transmissibility, duration of infectiousness, and immune evasion), lineage replacement dynamics are likely affected by spatiotemporal heterogeneity in incidence, NPIs, prior infection, and among-region mobility (*42*). The role of the latter may be enhanced in the context of low or declining prevalence, as suggested by the frequency growth of lineage B.1.177 in the UK and Europe during summer 2020, which was associated with international travel (*43*–*45*). Evidence for the increased intrinsic transmissibility of B.1.1.7 is clear, but estimates have varied considerably [38 to 130% increase (*7*, *9*)]. The growth potential of new SARS-CoV-2 variants will depend also on the average durations of their exposed and infectious phases, as well as their per-contact transmissibility (*36*). Our results indicate that exportations from a high-incidence epidemic source region raised early location-specific growth rate estimates across the UK (Fig. 4B), and that this effect declined through time. Similar trends have since been observed for lineage B.1.617.2 into the UK, after its importation from high-incidence regions onto a background of low incidence and lockdown easing. This conclusion is relevant for the interpretation of the current and future estimates of the increased transmissibility of B.1.1.7 (and other variants of concern) in other countries [such as the Untied States and Denmark (*3*)]. Further epidemiological and experimental work is needed to discriminate transient demographic factors from the permanent contribution to increased transmissibility conferred by the mutations carried by B.1.1.7.

Although B.1.1.7 was first detected in Kent, UK, and is speculated to have accumulated its mutations during a chronic infection (*2*), because of the strong correlation between human mobility from those areas and date of B.1.1.7 detection elsewhere our results support the hypothesis that B.1.1.7 originated in Kent or Greater London. Further, our phylogeographic reconstruction shows early lineage dissemination from Kent and Greater London, indicating that B.1.1.7 spread through the UK from one dominant UK source region, as opposed to a large undetected epidemic elsewhere, which would likely have resulted in multiple introductions through international travel (*16*).

We demonstrate that large-scale and well-sampled genomic surveillance data can reveal the detailed spatial transmission dynamics of individual SARS-CoV-2 lineages and compensate for their comparatively low genetic diversity (*46*). To achieve a representative genomic sample, we used only samples from population-level testing rather than those from specific outbreak investigations. However, this approach does not fully mitigate reduced representation from populations less likely to seek testing (*47*), and there is some geographic variation in the proportion of cases sequenced (fig. S15). Greater London consistently has a higher sampling proportion than other regions throughout the study timeframe. Although sampling biases cannot be wholly eliminated, the selection procedure used here, and our cross-validation between independent data sources (human mobility and SGTF datasets), help to ensure that our conclusions are robust. As SARS-CoV-2 genome sequencing efforts are accelerated worldwide, careful consideration and communication of sampling frameworks are needed to facilitate downstream epidemiological analyses (*48*). Spatial heterogeneity at the within-city scale was not accounted for in our analysis, consideration of which may further refine our understanding of the mechanisms of lineage emergence and invasion.

Coordinated and unified systems of genomic surveillance are needed worldwide to identify, track, and mitigate the transmission of SARS-CoV-2 variants of concern, including mechanisms to pair virus genomic and contact tracing data. Continuing rises in global incidence will increase the rate generation of viral genetic variation, and the accrual of higher levels of population immunity will create new selective pressures (*49*), the effects of which on virus evolution are difficult to predict (*50*–*52*). It is therefore critical to rapidly and accurately disentangle the contributions of genetic and ecological factors to the emergence of new SARS-CoV-2 variants. Geographic variation in vaccine availability, uptake, and delivery is expected to further contribute to variability in COVID-19 burden and the differential risk of disease resurgence (*17*, *53*, *54*), which can be mitigated through increased global access to vaccination and continued transmission control measures (*52*). Importation of SARS-CoV-2 lineages and variants from areas of high incidence will continue to pose a risk to those regions that are reducing NPIs after having controlled infection.

## Supplementary Material

20210722-1

## Supplementary Materials

science.sciencemag.org/content/373/6557/889/suppl/DC1
Materials and Methods Figs. S1 to S19 Tables S1 to S3 COVID-19 Genomics UK (CoG-UK) Consortium Author List References (*57*–*72*) MDAR Reproducibility Checklist
