# Supplementary material for: Spatiotemporal invasion dynamics of SARS-CoV-2 lineage B.1.1.7 emergence
Source: Science. 2021 Jul 22;373(6557):889–95. doi: 10.1126/science.abj0113 (PMC9269003; doi:10.1126/science.abj0113)
Supplement: 20210722-1 [file science.abj0113.v1.pdf]

Science

RESEARCH ARTICLES

Cite as: M. U. G. Kraemer *et al.*, *Science* 10.1126/science.abj0113 (2021).

Spatiotemporal invasion dynamics of SARS-CoV-2 lineage B.1.1.7 emergence

Moritz U. G. Kraemer[1]*†, Verity Hill[2]†, Christopher Ruis[3]†, Simon Dellicour[4,5]†, Sumali Bajaj[1]†, John T. McCrone[2], Guy Baele[5], Kris V. Parag[6], Anya Lindström Battle[7], Bernardo Gutierrez[1], Ben Jackson[2], Rachel Colquhoun[2], Áine O'Toole[2], Brennan Klein[8], Alessandro Vespignani[8], The COVID-19 Genomics UK (CoG-UK) consortium‡, Erik Volz[6], Nuno R. Faria[1,6,9], David Aanensen[10,11], Nicholas J. Loman[12], Louis du Plessis[1], Simon Cauchemez[13], Andrew Rambaut[2]*, Samuel V. Scarpino[8,14,15]*, Oliver G. Pybus[1,16]*

[1]Department of Zoology, University of Oxford, Oxford, UK. [2]Institute of Evolutionary Biology, University of Edinburgh, Edinburgh, UK. [3]Molecular Immunity Unit, Department of Medicine, Cambridge University, Cambridge, UK. [4]Spatial Epidemiology Lab (SpELL), Université Libre de Bruxelles, Bruxelles, Belgium. [5]Department of Microbiology, Immunology and Transplantation, Rega Institute, KU Leuven, 3000 Leuven, Belgium. [6]MRC Centre for Global Infectious Disease Analysis, Jameel Institute for Disease and Emergency Analytics, Imperial College London, London, UK. [7]Department of Plant Sciences, University of Oxford, Oxford, UK. [8]Network Science Institute, Northeastern University, Boston, USA. [9]Instituto de Medicina Tropical, Faculdade de Medicina da Universidade de Sao Paulo, Sao Paulo, Brazil. [10]Centre for Genomic Pathogen Surveillance, Wellcome Genome Campus, Hinxton, UK. [11]Big Data Institute, Li Ka Shing Centre for Health Information and Discovery, Nuffield Department of Medicine, University of Oxford, Oxford, UK. [12]Institute of Microbiology and Infection, University of Birmingham, Birmingham, UK. [13]Mathematical Modelling of Infectious Diseases Unit, Institut Pasteur, UMR2000, CNRS, Paris, France. [14]Vermont Complex Systems Center, University of Vermont, Burlington, USA. [15]Santa Fe Institute, Santa Fe, USA. [16]Department of Pathobiology and Population Sciences, Royal Veterinary College London, London, UK.

*Corresponding author. Email: a.rambaut@ed.ac.uk (A.R.); moritz.kraemer@zoo.ox.ac.uk (M.U.G.K.); s.scarpino@northeastern.edu (S.V.S.); oliver.pybus@zoo.ox.ac.uk (O.G.P.)

†These authors contributed equally to this work.

‡Consortium members and affiliations are listed in the supplementary materials.

Understanding the causes and consequences of the emergence of SARS-CoV-2 variants of concern is crucial to pandemic control yet difficult to achieve, as they arise in the context of variable human behavior and immunity. We investigate the spatial invasion dynamics of lineage B.1.1.7 by jointly analyzing UK human mobility, virus genomes, and community-based PCR data. We identify a multi-stage spatial invasion process in which early B.1.1.7 growth rates were associated with mobility and asymmetric lineage export from a dominant source location, enhancing the effects of B.1.1.7's increased intrinsic transmissibility. We further explore how B.1.1.7 spread was shaped by non-pharmaceutical interventions and spatial variation in previous attack rates. Our findings show that careful accounting of the behavioral and epidemiological context within which variants of concern emerge is necessary to interpret correctly their observed relative growth rates.

The SARS-CoV-2 lineage B.1.1.7 expanded rapidly across the United Kingdom (UK) (*1*, *2*) in late 2020 and subsequently spread internationally (*3*, *4*). As of 19 January 2021 (date of the most recent sample in our dataset), B.1.1.7 had reached all but five counties of Wales, Scotland, Northern Ireland and England, with onward transmission in each. Restrictions on international travel were enacted to contain B.1.1.7's spread, however genomic surveillance has since detected the presence and growth of the lineage in many countries worldwide (*4*, *5*). Analyses of genomic, laboratory, secondary contact, and aggregated epidemiological data estimate higher transmissibility of B.1.1.7 compared to previous SARS-CoV-2 lineages (*1*, *6*–*9*) and potentially a greater risk of hospitalisation (*10*–*13*). The spatial heterogeneity of SARS-CoV-2 transmission - and of emerging infectious diseases in general - can have profound effects on the local likelihood and intensity of transmission, final epidemic size, and immunity (*14*–*22*). More specifically, estimates of B.1.1.7's increased relative transmissibility declined during its emergence in the UK (*7*, *9*); understanding why this occurred is necessary if we are to respond effectively to future SARS-CoV-2 variants. Here, we reconstruct and quantify the spatial dynamics of B.1.1.7's emergence and investigate how human mobility and heterogeneity in previous exposure contributed to its initial spread and evaluation of higher transmissibility.

Spatial expansion and source sink dynamics of B.1.1.7 in the UK

B.1.1.7 can be first detected in CoG-UK genome data in Kent on 20 September 2020 and spread quickly across the

UK, with each week adding detections in approximately 7 new Upper Tier Local Authorities (UTLA) (table S2) (Fig. 1, A and B). Notably, B.1.1.7 was already reported in several UTLAs before the start of the second English lockdown (5 November 2020). By the end of that lockdown (2 December 2020), B.1.1.7 was widespread throughout the UK (Fig. 1, A and B).

The spatial expansion of SARS-CoV-2 lineages [e.g., (*16*, *24*)] can be tracked using data from the UK's national surveillance of SARS-CoV-2 genomes (*25*). By combining these data with aggregated mobile phone data, we examined the dissemination of B.1.1.7 via human mobility, from its likely location of emergence (Kent and Greater London) to other UK regions (Fig. 1, D and E, and materials and methods). Human mobility among UK regions increased at the end of the second English lockdown, from 55 to 75 million weekly movements (Fig. 1E). Due to its centrality, Greater London exhibits an important connective role in the UK human movement network (Fig. 1D, red lines represent the week the second lockdown was eased). Compared to previous weeks, movements out of Greater London were more frequent and reached more unique destinations (fig. S1). For each UTLA, we find that the date of first detection of B.1.1.7 is predicted well by human mobility from Kent and Greater London to that UTLA (Pearson's r = −0.73, 95% CI: −0.61, −0.81, Fig. 1C; AIC = 734) and similarly well using movements from Kent and Greater London separately (fig. S2). This correlation strengthens through time as new locations of B.1.1.7 detection are added (fig. S3) and is robust to changes in human mobility through time in among-region human movement (Pearson's r = −0.44, 95% CI: −0.16, −0.65, P value < 0.01, mobility data through 23 January 2021; materials and methods). Geographic distance from Greater London correlates less strongly with B.1.1.7 arrival times (Pearson's r = 0.60, 95% CI: 0.44 - 0.71; AIC = 763, fig. S4).

To understand better the spatial dispersal of B.1.1.7 during its emergence, we reconstructed its spread across England using large-scale phylogeographic analysis (*26*–*28*). We analyzed 17,716 B.1.1.7 genomes collected between 20 September 2020 and 19 January 2021 (Fig. 2 and fig. S5), collated from PCR-positive community samples that represent a random selection of SARS-CoV-2-positive samples (*29*). These genomes represent ~4% of UK B.1.1.7 cases during the study period (n = 460,510 estimated tests with PCR S-gene target failure, SGTF, between 20 September 2020 and 19 January 2021). Samples per location (UTLA) and per week in the SGTF and whole genome datasets are strongly correlated [Pearson's r = 0.69, 95% CI: 0.63 – 0.73, P value < 0.001, fig. S6, (*7*)], making it feasible to reconstruct B.1.1.7 expansion history using phylogeographic approaches (*30*).

We identify distinct phases to the emergence of B.1.1.7. Initially, during the second English lockdown, most (71.2%) B.1.1.7 phylogenetic branch movements originated and ended in Greater London/Kent; long distance dispersal events were relatively infrequent (Figs. 2 and 3). After the lockdown ended, and new cases in London subsequently rose rapidly, observed virus lineage movements from southeast England to other regions increased and other large cities started to exhibit local transmission (Figs. 2 and 3). This phase of growing number of exported B.1.1.7 cases from London and environs stabilized in mid-December and coincided with reduced mobility from Greater London (Tier 4 restrictions were announced on December 20, 2020 and entailed a "Stay at home" order, closure of non-essential shops and hospitality, and strict limitations on household mixing, Figs. 1E and 2C). However, the total number of B.1.1.7 lineage exports did not immediately decline, because the growing number of B.1.1.7 cases in southeast England offset the decline in outward travel (Fig. 2C) (*31*), indicating a limited effect of delayed action on B.1.1.7 spread from Greater London. Our analysis did not allow us to establish a causal link between NPIs and their impact on lineage exportations so these results should be interpreted with caution.

By combining mobility and SGTF data with estimates of the proportion of the population testing SARS-CoV-2-positive (materials and methods) we can estimate the frequency of B.1.1.7 export from Greater London to other English regions (Fig. 2C and fig. S7) and explore its role in accelerating the lineage's emergence. Using these combined data sources, we estimate the number of B.1.1.7 case exports from Greater London rose during November (including during lockdown) from <600 to >12,000 in early December (Fig. 2C, grey curve), reflecting growth in B.1.1.7 infections in Greater London and an increase in human mobility among UK geographic regions across in late November (Fig. 1E). The estimated intensity of B.1.1.7 case exportation from Greater London remained high in December, peaking in mid-December at ~20,000 weekly exports, before declining in early January after the third national lockdown started on 5 January 2021. Importantly, these estimates (Fig. 2C, grey curve) closely match the trends in lineage B.1.1.7 movement inferred from phylogeographic analysis (Fig. 2C, red curve), cross-validating both data sources (exports estimated using each method are strongly correlated; Pearson's r = 0.62, 95% CI: 0.61 – 0.64, P value < 0.001, fig. S8). Lineage exportation events estimated from genomic data are lower from late December onwards, possibly due to reporting lags in genomic data generation and/or delayed care-seeking due to the Christmas holidays (*32*). Our simple model assumes that non-symptomatic infectious individuals are equally likely to travel (Fig. 2C, grey line), which may bias our estimates of infectious travellers upwards.

B.1.1.7 dispersal dynamics shifted in late December to more bi-directional exchange of phylogenetic lineages in and out of Greater London (Fig. 3), coinciding with rapid growth in B.1.1.7 cases across England (*9*). Throughout, the weekly

number of B.1.1.7 cases in a UTLA was positively associated with the number of B.1.1.7 lineage introductions into that UTLA during that week (Pearson's r = 0.41, 0.76, 0.91, and 0.73, for October, November, December and January; P < 0.001 for all; fig. S6; see supplementary materials for further analysis). We observe spatial heterogeneity in B.1.1.7 lineage importations; in the phylogeographic analysis some locations received >500 inferred importations, despite the fact that our genomic dataset represents <4% of reported B.1.1.7 cases during the study period (Fig. 2D).

Detailed mapping of the spatial dynamics of SARS-CoV-2 lineages is difficult without comprehensive, well-sampled epidemiological and genomic data (*33*, *34*). However, the CoG-UK data enables us to study dissemination trends by comparing inferred B.1.1.7 importations to within-location movements. Greater London (and to some extent Kent) acted as the main exporter of B.1.1.7 lineages to other UTLAs until mid-December 2020 (Fig. 3A). The longest (>100km) and shortest (<100km) dispersal events consistently originated from Greater London throughout the study period (Fig. 3B), primarily due to its large epidemic. However, the relative percentage of lineage movements that originated from Greater London approximately halved between September 2020 and January 2021; table S1).

Spatial heterogeneity in SARS-CoV-2 incidence and B.1.1.7 expansion

Using SGTF PCR-positive tests as a proxy for B.1.1.7 infection (*35*), we next examined daily growth rates of SARS-CoV-2 cases at the UTLA level for SGTF and non-SGTF cases (*36*) (excluding case data from 25–31 January to account for reporting and testing delays; materials and methods). Case growth rates immediately following the November 2020 lockdown were highest in regions of southeast England connected to Greater London/Kent (fig. S9). Acceleration in SGTF case growth rates in Greater London began in mid-November and preceded acceleration in other regions (Fig. 4B). At the UTLA level, growth rates of SGTF cases were significantly higher than non-SGTF cases (fig. S9), a key observation used to support an increased transmissibility for B.1.1.7 (*7*, *9*)

Here, we add to those findings by quantifying the import of B.1.1.7 cases from London and investigate the association of importation trends with lineage-specific case growth rates (materials and methods). Using our phylogeographic analysis results (Figs. 2 and 3) we find that growth in the rate of B.1.1.7 importation into a LTLA closely matches the early growth rate of SGTF cases in that LTLA (Fig. 4A shows Birmingham, Liverpool and Manchester). We further calculated the per-region difference between SGTF and non-SGTF case growth rates [fig. S11; the estimated raw additive increase in SGTF growth rate - is 0.0715 and median multiplicative advantage is 1.576, assuming a generation time of 6.5 days); qualitatively similar to those reported previously (*7*, *9*), with the caveat that generation times may differ between B.1.1.7 and other lineages (*37*, *38*)]. The degree to which this difference is positively correlated with B.1.1.7 importation rate grew during the latter half of the November lockdown and remained remarkably high (R^2 > 0.75) until mid December, before declining (Fig. 4C; the trend remains when accounting for uncertainty in the estimated number of infections across Greater London, figs. S12). This result is robust to the data and methods used to estimate per-location B.1.1.7 importation rates (figs. S9 and S10). Accounting for continued export of B.1.1.7 from Greater London and Kent can explain in part why estimates of the growth advantage of B.1.1.7 declined during the second half of December 2020, before the implementation of tighter control measures (Tier 4, December 20) (*7*, *9*).

Human mobility and prior outbreaks as predictors of B.1.1.7 growth

The epicenter of SARS-CoV-2 transmission in the UK shifted during the November 2020 lockdown: between 1 September and 1 December 2020, ~80% of reported cases were reported outside London and southeast England, while those regions accounted for ~40% of all cases during December 1–7. We sought to understand how, in each location, post-lockdown growth rates related to previous attack rates as well as travel inflow to that location. We investigated predictors of the increase in the relative frequency of B.1.1.7 genomes compared to that of other SARS-CoV-2 lineages (Fig. 5A) (*7*, *9*). In a multivariate model, we find that about half of the variation in the increase in B.1.1.7 relative frequency between 2 and 16 December is associated with human mobility from Greater London and attack rates prior to the November lockdown (Fig. 5, B and C). UTLAs with lower previous attack rates tended to have faster-increasing B.1.1.7 frequencies. We repeated this analysis using SGTF case frequency data and obtained similar results (R^2 = 0.57, P < 0.001) (fig. S13). However, neither human mobility nor pre-lockdown attack rate were significant predictors of later changes. Instead, change in the relative frequency of B.1.1.7 genomes after 17 December was best predicted simply by its frequency on that date (R^2 = 0.13, P < 0.01; fig. S14). Although we note that a model identified through exhaustive search using BIC includes the "frequency of B.1.1.7 on December 17", an interaction between arrival time and "frequency of B.1.1.7 on December 17" and an interaction between incidence prior to the November lockdown and mobility from London (BIC 178.467, R^2 = 0.68, P < 0.001, fig. S14). Mobility from Greater London remains a significant predictor of B.1.1.7 growth after controlling for population size using both a multivariate regression and model-selection using exhaustive search with both BIC and AIC.

Conclusions, limitations and future work

We find the emergence of B.1.1.7 throughout the UK was associated with a high export frequency from a major source location that was identified only retrospectively. This pattern recapitulates at a national scale the role that international mobility played in the early spread of the SARS-CoV-2 pandemic (*39–41*). We conclude that the exceptionally rapid spatial spread and early growth rates of lineage B.1.1.7 likely reflect the combined effects of its higher intrinsic transmissibility (*1*, *7*, *9*) and the spatial structure of incidence and mobility before, during, and after the second lockdown in England (*42*).

Understanding what causes a new SARS-CoV-2 lineage to grow and replace pre-existing lineages is a complex problem. In addition to virus genetic changes to relevant phenotypes (such as per-contact transmissibility, duration of infectiousness, and immune evasion) lineage replacement dynamics are likely affected by spatio-temporal heterogeneity in incidence, NPIs, prior infection, and among-region mobility (*43*). The role of the latter may be enhanced in the context of low or declining prevalence, as suggested by the frequency growth of lineage B.1.177 in the UK and Europe during summer 2020, which was associated with international travel (*44–46*). Evidence for the increased intrinsic transmissibility of B.1.1.7 is clear but estimates have varied considerably [38–130% increase (*7*, *9*)]. The growth potential of new SARS-CoV-2 variants will depend also on the average durations of their exposed and infectious phases, as well as their per-contact transmissibility (*37*). Our results indicate that exportations from a high-incidence epidemic source region raised early location-specific growth rate estimates across the UK (Fig. 4B), and that this effect declined through time. Similar trends have since been observed for lineage B.1.617.2 into the UK, following its importation from high incidence regions onto a background of low incidence and lockdown easing. This conclusion is important for the interpretation of the current and future estimates of the increased transmissibility of B.1.1.7 (and other variants of concern) in other countries [e.g., USA, Denmark (*3*)]. Further epidemiological and experimental work is needed to discriminate transient demographic factors from the permanent contribution to increased transmissibility conferred by the mutations carried by B.1.1.7.

Although B.1.1.7 was first detected in Kent, UK, and is speculated to have accumulated its mutations during a chronic infection (*47*), our results support the hypothesis that B.1.1.7 originated in Kent or Greater London, due to the strong correlation between human mobility from those areas and date of B.1.1.7 detection elsewhere. Further, our phylogeographic reconstruction shows early lineage dissemination from Kent and Greater London, indicating that B.1.1.7 spread through the UK from one dominant UK source region, as opposed to a large undetected epidemic elsewhere, which would likely have resulted in multiple introductions through international travel (*16*).

We demonstrate that large-scale and well-sampled genomic surveillance data can reveal the detailed spatial transmission dynamics of individual SARS-CoV-2 lineages, and compensate for their comparatively low genetic diversity (*48*). To achieve a representative genomic sample, we used only samples from population-level testing rather than those from specific outbreak investigations. However, this approach does not fully mitigate reduced representation from populations less likely to seek testing (*49*) and there is some geographic variation in the proportion of cases sequenced (fig. S15). We note that Greater London consistently has a higher sampling proportion than other regions throughout the study timeframe. Although sampling biases cannot be wholly eliminated, the selection procedure used here, and our cross-validation between independent data sources (human mobility and SGTF datasets), help to ensure our conclusions are robust. As SARS-CoV-2 genome sequencing efforts are accelerated worldwide, careful consideration and communication of sampling frameworks are needed to facilitate downstream epidemiological analyses (*50*). Spatial heterogeneity at the within-city scale was not accounted for in our analysis, consideration of which may further refine our understanding of the mechanisms of lineage emergence and invasion.

Coordinated and unified systems of genomic surveillance are needed worldwide to identify, track, and mitigate the transmission of SARS-CoV-2 variants of concern, including mechanisms to pair virus genomic and contact tracing data. Continuing rises in global incidence will increase the rate generation of viral genetic variation, while the accrual of higher levels of population immunity will create new selective pressures (*51*), the effects of which on virus evolution are difficult to predict (*52–54*). It is therefore critical to rapidly and accurately disentangle the contribution of genetic and ecological factors to the emergence of new SARS-CoV-2 variants. Geographic variation in vaccine availability, uptake and delivery is expected to further contribute to variability in COVID-19 burden and the differential risk of disease resurgence (*17*, *55*, *56*), which can be mitigated by increased global access to vaccination and continued transmission control measures (*54*). Importation of SARS-CoV-2 lineages and variants from areas of high incidence will continue to pose a risk to those regions that are reducing NPIs after having controlled infection.

REFERENCES AND NOTES

1. M. Chand, S. Hopkins, C. Achison, C. Anderson, H. Allen, P. Blomquist, C. Chen, V. Chalker, G. Dabrera, O. Edeghere, M. Edmunds, T. Lamagni, R. Myers, I. Oliver, R. Elson, E. Gallagher, N. Groves, G. Hughes, M. Kall, H. Moore, W. Sopwith, C. Turner, L. Utsi, M. Vabistsevits, R. Vivancos, A. Zaidi, M. Zambon, W. Barclay, N. Ferguson, E. Volz, N. Loman, A. Rambaut, J. Barrett, Investigation of novel SARS-CoV-2 variant Variant of Concern 202012/01 (2021); https://assets.publishing.service.gov.uk/government/uploads/system/uploads/attachment_data/file/952490/Variant_of_Concern_VOC_202012_01_Technical_Briefing_4_England.pdf.
2. COVID-19 Genomics Consortium UK (CoG-UK), Preliminary genomic characterisation of an emergent SARS-CoV-2 lineage in the UK defined by a novel set of spike mutations (2020); https://virological.org/t/preliminary-genomic-characterisation-of-an-emergent-sars-cov-2-lineage-in-the-uk-defined-by-a-novel-set-of-spike-mutations/563.
3. T. Alpert, A. F. Brito, E. Lasek-Nesselquist, J. Rothman, A. L. Valesano, M. J. MacKay, M. E. Petrone, M. I. Breban, A. E. Watkins, C. B. F. Vogels, C. C. Kalinich, S. Dellicour, A. Russell, J. P. Kelly, M. Shudt, J. Plitnick, E. Schneider, W. J. Fitzsimmons, G. Khullar, J. Metti, J. T. Dudley, M. Nash, N. Beaubier, J. Wang, C. Liu, P. Hui, A. Muyombwe, R. Downing, J. Razeq, S. M. Bart, A. Grills, S. M. Morrison, S. Murphy, C. Neal, E. Laszlo, H. Rennert, M. Cushing, L. Westblade, P. Velu, A. Craney, L. Cong, D. R. Peaper, M. L. Landry, P. W. Cook, J. R. Fauver, C. E. Mason, A. S. Lauring, K. St George, D. R. MacCannell, N. D. Grubaugh, Early introductions and transmission of SARS-CoV-2 variant B.1.1.7 in the United States. *Cell* **184**, 2595–2604.e13 (2021). doi:10.1016/j.cell.2021.03.061 Medline
4. Á. O'Toole, V. Hill, O. G. Pybus, A. Watts, I. I. Bogoch, K. Khan, J. P. Messina, H. Tegally, R. R. Lessells, J. Giandhari, S. Pillay, K. A. Tumedi, G. Nyepetsi, M. Kebabonye, M. Matsheka, M. Mine, S. Tokajian, H. Hassan, T. Salloum, G. Merhi, J. Koweyes, J. L. Geoghegan, J. de Ligt, X. Ren, M. Storey, N. E. Freed, C. Pattabiraman, P. Prasad, A. S. Desai, R. Vasanthapuram, T. F. Schulz, L. Steinbrück, T. Stadler, A. Parisi, A. Bianco, D. García de Viedma, S. Buenestado-Serrano, V. Borges, J. Isidro, S. Duarte, J. P. Gomes, N. S. Zuckerman, M. Mandelboim, O. Mor, T. Seemann, A. Arnott, J. Draper, M. Gall, W. Rawlinson, I. Deveson, S. Schlebusch, J. McMahon, L. Leong, C. K. Lim, M. Chironna, D. Loconsole, A. Bal, L. Josset, E. Holmes, K. St George, E. Lasek-Nesselquist, R. S. Sikkema, B. Oude Munnink, M. Koopmans, M. Brytting, V. Sudha Rani, S. Pavani, T. Smura, A. Heim, S. Kurkela, M. Umair, M. Salman, B. Bartolini, M. Rueca, C. Drosten, T. Wolff, O. Silander, D. Eggink, C. Reusken, H. Vennema, A. Park, C. Carrington, N. Sahadeo, M. Carr, G. Gonzalez, T. de Oliveira, N. Faria, A. Rambaut, M. U. G. Kraemer; COVID-19 Genomics UK (COG-UK) consortium; Network for Genomic Surveillance in South Africa (NGS-SA); Brazil-UK CADDE Genomic Network; Swiss Viollier Sequencing Consortium; SEARCH Alliance San Diego; National Virus Reference Laboratory; SeqCOVID-Spain; Danish Covid-19 Genome Consortium (DCGC); Communicable Diseases Genomic Network (CDGN); Dutch National SARS-CoV-2 surveillance program; Division of Emerging Infectious Diseases (KDCA), Tracking the international spread of SARS-CoV-2 lineages B.1.1.7 and B.1.351/501Y-V2. *Wellcome Open Res.* **6**, 121 (2021). doi:10.12688/wellcomeopenres.16661.1 Medline
5. N. L. Washington, K. Gangavarapu, M. Zeller, A. Bolze, E. T. Cirulli, K. M. Schiabor Barrett, B. B. Larsen, C. Anderson, S. White, T. Cassens, S. Jacobs, G. Levan, J. Nguyen, J. M. Ramirez 3rd, C. Rivera-Garcia, E. Sandoval, X. Wang, D. Wong, E. Spencer, R. Robles-Sikisaka, E. Kurzban, L. D. Hughes, X. Deng, C. Wang, V. Servellita, H. Valentine, P. De Hoff, P. Seaver, S. Sathe, K. Gietzen, B. Sickler, J. Antico, K. Hoon, J. Liu, A. Harding, O. Bakhtar, T. Basler, B. Austin, D. MacCannell, M. Isaksson, P. G. Febbo, D. Becker, M. Laurent, E. McDonald, G. W. Yeo, R. Knight, L. C. Laurent, E. de Feo, M. Worobey, C. Y. Chiu, M. A. Suchard, J. T. Lu, W. Lee, K. G. Andersen, Emergence and rapid transmission of SARS-CoV-2 B.1.1.7 in the United States. *Cell* **184**, 2587–2594.e7 (2021). doi:10.1016/j.cell.2021.03.052 Medline
6. Lineage-specific growth of SARS-CoV-2 B.1.1.7 during the English national lockdown (2020); https://virological.org/t/lineage-specific-growth-of-sars-cov-2-b-1-1-7-during-the-english-national-lockdown/575.
7. E. Volz, S. Mishra, M. Chand, J. C. Barrett, R. Johnson, L. Geidelberg, W. R. Hinsley, D. J. Laydon, G. Dabrera, Á. O'Toole, R. Amato, M. Ragonnet-Cronin, I. Harrison, B. Jackson, C. V. Ariani, O. Boyd, N. J. Loman, J. T. McCrone, S. Gonçalves, D. Jorgensen, R. Myers, V. Hill, D. K. Jackson, K. Gaythorpe, N. Groves, J. Sillitoe, D. P. Kwiatkowski, S. Flaxman, O. Ratmann, S. Bhatt, S. Hopkins, A. Gandy, A. Rambaut, N. M. Ferguson; COVID-19 Genomics UK (COG-UK) consortium, Assessing transmissibility of SARS-CoV-2 lineage B.1.1.7 in England. *Nature* **593**, 266–269 (2021). doi:10.1038/s41586-021-03470-x Medline
8. T. C. Jones, G. Biele, B. Mühlemann, T. Veith, J. Schneider, J. Beheim-Schwarzbach, T. Bleicker, J. Tesch, M. L. Schmidt, L. E. Sander, F. Kurth, P. Menzel, R. Schwarzer, M. Zuchowski, J. Hofmann, A. Krumbholz, A. Stein, A. Edelmann, V. M. Corman, C. Drosten, Estimating infectiousness throughout SARS-CoV-2 infection course. *Science* **373**, eabi5273 (2021). doi:10.1126/science.abi5273 Medline
9. N. G. Davies, S. Abbott, R. C. Barnard, C. I. Jarvis, A. J. Kucharski, J. D. Munday, C. A. B. Pearson, T. W. Russell, D. C. Tully, A. D. Washburne, T. Wenseleers, A. Gimma, W. Waites, K. L. M. Wong, K. van Zandvoort, J. D. Silverman, K. Diaz-Ordaz, R. Keogh, R. M. Eggo, S. Funk, M. Jit, K. E. Atkins, W. J. Edmunds; CMMID COVID-19 Working Group; COVID-19 Genomics UK (COG-UK) Consortium, Estimated transmissibility and impact of SARS-CoV-2 lineage B.1.1.7 in England. *Science* **372**, eabg3055 (2021). doi:10.1126/science.abg3055 Medline
10. R. Challen, E. Brooks-Pollock, J. M. Read, L. Dyson, K. Tsaneva-Atanasova, L. Danon, Risk of mortality in patients infected with SARS-CoV-2 variant of concern 202012/1: Matched cohort study. *BMJ* **372**, n579 (2021). doi:10.1136/bmj.n579 Medline
11. Scientific Advisory Group for Emergencies, NERVTAG: Update note on B.1.1.7 severity, 11 February 2021 (2021); https://www.gov.uk/government/publications/nervtag-update-note-on-b117-severity-11-february-2021.
12. N. G. Davies, C. I. Jarvis, W. J. Edmunds, N. P. Jewell, K. Diaz-Ordaz, R. H. Keogh; CMMID COVID-19 Working Group, Increased mortality in community-tested cases of SARS-CoV-2 lineage B.1.1.7. *Nature* **593**, 270–274 (2021). doi:10.1038/s41586-021-03426-1 Medline
13. D. Frampton, T. Rampling, A. Cross, H. Bailey, J. Heaney, M. Byott, R. Scott, R. Sconza, J. Price, M. Margaritis, M. Bergstrom, M. J. Spyer, P. B. Miralhes, P. Grant, S. Kirk, C. Valerio, Z. Mangera, T. Prabhahar, J. Moreno-Cuesta, N. Arulkumaran, M. Singer, G. Y. Shin, E. Sanchez, S. M. Paraskevopoulou, D. Pillay, R. A. McKendry, M. Mirfenderesky, C. F. Houlihan, E. Nastouli, Genomic characteristics and clinical effect of the emergent SARS-CoV-2 B.1.1.7 lineage in London, UK: A whole-genome sequencing and hospital-based cohort study. *Lancet Infect. Dis.* S1473-3099(21)00170-5 (2021). doi:10.1016/S1473-3099(21)00170-5 Medline
14. A. Rambaut, O. G. Pybus, M. I. Nelson, C. Viboud, J. K. Taubenberger, E. C. Holmes, The genomic and epidemiological dynamics of human influenza A virus. *Nature* **453**, 615–619 (2008). doi:10.1038/nature06945 Medline
15. C. Viboud, O. N. Bjørnstad, D. L. Smith, L. Simonsen, M. A. Miller, B. T. Grenfell, Synchrony, waves, and spatial hierarchies in the spread of influenza. *Science* **312**, 447–451 (2006). doi:10.1126/science.1125237 Medline
16. L. du Plessis, J. T. McCrone, A. E. Zarebski, V. Hill, C. Ruis, B. Gutierrez, J. Raghwani, J. Ashworth, R. Colquhoun, T. R. Connor, N. R. Faria, B. Jackson, N. J. Loman, Á. O'Toole, S. M. Nicholls, K. V. Parag, E. Scher, T. I. Vasylyeva, E. M. Volz, A. Watts, I. I. Bogoch, K. Khan, D. M. Aanensen, M. U. G. Kraemer, A. Rambaut, O. G. Pybus; COVID-19 Genomics UK (COG-UK) Consortium, Establishment and lineage dynamics of the SARS-CoV-2 epidemic in the UK. *Science* **371**, 708–712 (2021). doi:10.1126/science.abf2946 Medline
17. R. M. May, R. M. Anderson, Spatial heterogeneity and the design of immunization programs. *Math. Biosci.* **72**, 83–111 (1984). doi:10.1016/0025-5564(84)90063-4
18. D. J. Watts, R. Muhamad, D. C. Medina, P. S. Dodds, Multiscale, resurgent epidemics in a hierarchical metapopulation model. *Proc. Natl. Acad. Sci. U.S.A.* **102**, 11157–11162 (2005). doi:10.1073/pnas.0501226102 Medline
19. C. J. E. Metcalf, C. V. Munayco, G. Chowell, B. T. Grenfell, O. N. Bjørnstad, Rubella metapopulation dynamics and importance of spatial coupling to the risk of congenital rubella syndrome in Peru. *J. R. Soc. Interface* **8**, 369–376 (2011). doi:10.1098/rsif.2010.0320 Medline
20. B. D. Dalziel, S. Kissler, J. R. Gog, C. Viboud, O. N. Bjørnstad, C. J. E. Metcalf, B. T. Grenfell, Urbanization and humidity shape the intensity of influenza epidemics in U.S. cities. *Science* **362**, 75–79 (2018). doi:10.1126/science.aat6030 Medline

21. M. S. Y. Lau, A. D. Becker, H. M. Korevaar, Q. Caudron, D. J. Shaw, C. J. E. Metcalf, O. N. Bjørnstad, B. T. Grenfell, A competing-risks model explains hierarchical spatial coupling of measles epidemics en route to national elimination. *Nat. Ecol. Evol.* **4**, 934–939 (2020). doi:10.1038/s41559-020-1186-6 Medline
22. G. Chowell, L. Sattenspiel, S. Bansal, C. Viboud, Mathematical models to characterize early epidemic growth: A review. *Phys. Life Rev.* **18**, 66–97 (2016). doi:10.1016/j.plrev.2016.07.005 Medline
23. Tier 4 Restrictions for London, Bedfordshire and Luton; https://www.elft.nhs.uk/News/Tier-4-Restrictions-for-London-Bedfordshire-and-Luton.
24. D. S. Candido, I. M. Claro, J. G. de Jesus, W. M. Souza, F. R. R. Moreira, S. Dellicour, T. A. Mellan, L. du Plessis, R. H. M. Pereira, F. C. S. Sales, E. R. Manuli, J. Thézé, L. Almeida, M. T. Menezes, C. M. Voloch, M. J. Fumagalli, T. M. Coletti, C. A. M. da Silva, M. S. Ramundo, M. R. Amorim, H. H. Hoeltgebaum, S. Mishra, M. S. Gill, L. M. Carvalho, L. F. Buss, C. A. Prete Jr., J. Ashworth, H. I. Nakaya, P. S. Peixoto, O. J. Brady, S. M. Nicholls, A. Tanuri, Á. D. Rossi, C. K. V. Braga, A. L. Gerber, A. P. de C Guimarães, N. Gaburo Jr., C. S. Alencar, A. C. S. Ferreira, C. X. Lima, J. E. Levi, C. Granato, G. M. Ferreira, R. S. Francisco Jr., F. Granja, M. T. Garcia, M. L. Moretti, M. W. Perroud Jr., T. M. P. P. Castiñeiras, C. S. Lazari, S. C. Hill, A. A. de Souza Santos, C. L. Simeoni, J. Forato, A. C. Sposito, A. Z. Schreiber, M. N. N. Santos, C. Z. de Sá, R. P. Souza, L. C. Resende-Moreira, M. M. Teixeira, J. Hubner, P. A. F. Leme, R. G. Moreira, M. L. Nogueira, N. M. Ferguson, S. F. Costa, J. L. Proenca-Modena, A. T. R. Vasconcelos, S. Bhatt, P. Lemey, C.-H. Wu, A. Rambaut, N. J. Loman, R. S. Aguiar, O. G. Pybus, E. C. Sabino, N. R. Faria; Brazil-UK Centre for Arbovirus Discovery, Diagnosis, Genomics and Epidemiology (CADDE) Genomic Network, Evolution and epidemic spread of SARS-CoV-2 in Brazil. *Science* **369**, 1255–1260 (2020). doi:10.1126/science.abd2161 Medline
25. COVID-19 Genomics UK (COG-UK) consortiumcontact@cogconsortium.uk, An integrated national scale SARS-CoV-2 genomic surveillance network. *Lancet Microbe* **1**, e99–e100 (2020). doi:10.1016/S2666-5247(20)30054-9 Medline
26. P. Lemey, A. Rambaut, J. J. Welch, M. A. Suchard, Phylogeography takes a relaxed random walk in continuous space and time. *Mol. Biol. Evol.* **27**, 1877–1885 (2010). doi:10.1093/molbev/msq067 Medline
27. O. G. Pybus, M. A. Suchard, P. Lemey, F. J. Bernardin, A. Rambaut, F. W. Crawford, R. R. Gray, N. Arinaminpathy, S. L. Stramer, M. P. Busch, E. L. Delwart, Unifying the spatial epidemiology and molecular evolution of emerging epidemics. *Proc. Natl. Acad. Sci. U.S.A.* **109**, 15066–15071 (2012). doi:10.1073/pnas.1206598109 Medline
28. S. Dellicour, K. Durkin, S. L. Hong, B. Vanmechelen, J. Martí-Carreras, M. S. Gill, C. Meex, S. Bontems, E. André, M. Gilbert, C. Walker, N. Maio, N. R. Faria, J. Hadfield, M.-P. Hayette, V. Bours, T. Wawina-Bokalanga, M. Artesi, G. Baele, P. Maes, A phylodynamic workflow to rapidly gain insights into the dispersal history and dynamics of SARS-CoV-2 lineages. *Mol. Biol. Evol.* **38**, 1608–1613 (2021). doi:10.1093/molbev/msaa284 Medline
29. COVID-19 testing data: methodology note; https://www.gov.uk/government/publications/coronavirus-covid-19-testing-data-methodology/covid-19-testing-data-methodology-note.
30. A. Kalkauskas, U. Perron, Y. Sun, N. Goldman, G. Baele, S. Guindon, N. De Maio, Sampling bias and model choice in continuous phylogeography: Getting lost on a random walk. *PLOS Comput. Biol.* **17**, e1008561 (2021). doi:10.1371/journal.pcbi.1008561 Medline
31. S. Francis, Covid: Christmas Tier-4 heartbreak for Londoners. *BBC* (2020); at https://www.bbc.co.uk/news/uk-england-london-55380644.
32. Official UK Coronavirus Dashboard; https://coronavirus.data.gov.uk/details/testing?areaType=nation%26areaName=England.
33. J. Bahl, M. I. Nelson, K. H. Chan, R. Chen, D. Vijaykrishna, R. A. Halpin, T. B. Stockwell, X. Lin, D. E. Wentworth, E. Ghedin, Y. Guan, J. S. M. Peiris, S. Riley, A. Rambaut, E. C. Holmes, G. J. D. Smith, Temporally structured metapopulation dynamics and persistence of influenza A H3N2 virus in humans. *Proc. Natl. Acad. Sci. U.S.A.* **108**, 19359–19364 (2011). doi:10.1073/pnas.1109314108 Medline
34. G. Dudas, L. M. Carvalho, T. Bedford, A. J. Tatem, G. Baele, N. R. Faria, D. J. Park, J. T. Ladner, A. Arias, D. Asogun, F. Bielejec, S. L. Caddy, M. Cotten, J. D'Ambrozio, S. Dellicour, A. Di Caro, J. W. Diclaro, S. Duraffour, M. J. Elmore, L. S. Fakoli, O. Faye, M. L. Gilbert, S. M. Gevao, S. Gire, A. Gladden-Young, A. Gnirke, A. Goba, D. S. Grant, B. L. Haagmans, J. A. Hiscox, U. Jah, J. R. Kugelman, D. Liu, J. Lu, C. M. Malboeuf, S. Mate, D. A. Matthews, C. B. Matranga, L. W. Meredith, J. Qu, J. Quick, S. D. Pas, M. V. T. Phan, G. Pollakis, C. B. Reusken, M. Sanchez-Lockhart, S. F. Schaffner, J. S. Schieffelin, R. S. Sealfon, E. Simon-Loriere, S. L. Smits, K. Stoecker, L. Thorne, E. A. Tobin, M. A. Vandi, S. J. Watson, K. West, S. Whitmer, M. R. Wiley, S. M. Winnicki, S. Wohl, R. Wölfel, N. L. Yozwiak, K. G. Andersen, S. O. Blyden, F. Bolay, M. W. Carroll, B. Dahn, B. Diallo, P. Formenty, C. Fraser, G. F. Gao, R. F. Garry, I. Goodfellow, S. Günther, C. T. Happi, E. C. Holmes, B. Kargbo, S. Keïta, P. Kellam, M. P. G. Koopmans, J. H. Kuhn, N. J. Loman, N. Magassouba, D. Naidoo, S. T. Nichol, T. Nyenswah, G. Palacios, O. G. Pybus, P. C. Sabeti, A. Sall, U. Ströher, I. Wurie, M. A. Suchard, P. Lemey, A. Rambaut, Virus genomes reveal factors that spread and sustained the Ebola epidemic. *Nature* **544**, 309–315 (2017). doi:10.1038/nature22040 Medline
35. Public Health England, Investigation of novel SARS-CoV-2 variants of concern (2020); https://www.gov.uk/government/publications/investigation-of-novel-sars-cov-2-variant-variant-of-concern-20201201.
36. S. A. Kemp, R. P. Datir, D. A. Collier, I. Ferreira, A. Carabelli, W. Harvey, D. L. Robertson, R. K. Gupta, Recurrent emergence and transmission of a SARS-CoV-2 Spike deletion ΔH69/ΔV70. bioRxiv [Preprint] 15 December 2020). doi:10.1101/2020.12.14.422555.
37. S. W. Park, B. M. Bolker, S. Funk, C. J. E. Metcalf, J. S. Weitz, B. T. Grenfell, J. Dushoff, Roles of generation-interval distributions in shaping relative epidemic strength, speed, and control of new SARS-CoV-2 variants. bioRxiv [Preprint] 5 May 2021. doi:10.1101/2021.05.03.21256545.
38. W. S. Hart, S. Abbott, A. Endo, J. Hellewell, E. Miller, N. Andrews, P. K. Maini, S. Funk, R. N. Thompson, Inference of SARS-CoV-2 generation times using UK household data. medRxiv [Preprint] 30 May 2021. doi:10.1101/2021.05.27.21257936.
39. M. Chinazzi, J. T. Davis, M. Ajelli, C. Gioannini, M. Litvinova, S. Merler, A. Pastore Y Piontti, K. Mu, L. Rossi, K. Sun, C. Viboud, X. Xiong, H. Yu, M. E. Halloran, I. M. Longini Jr., A. Vespignani, The effect of travel restrictions on the spread of the 2019 novel coronavirus (COVID-19) outbreak. *Science* **368**, 395–400 (2020). doi:10.1126/science.aba9757 Medline
40. M. Worobey, J. Pekar, B. B. Larsen, M. I. Nelson, V. Hill, J. B. Joy, A. Rambaut, M. A. Suchard, J. O. Wertheim, P. Lemey, The emergence of SARS-CoV-2 in Europe and North America. *Science* **370**, 564–570 (2020). doi:10.1126/science.abc8169 Medline
41. A. S. Gonzalez-Reiche, M. M. Hernandez, M. J. Sullivan, B. Ciferri, H. Alshammary, A. Obla, S. Fabre, G. Kleiner, J. Polanco, Z. Khan, B. Alburquerque, A. van de Guchte, J. Dutta, N. Francoeur, B. S. Melo, I. Oussenko, G. Deikus, J. Soto, S. H. Sridhar, Y.-C. Wang, K. Twyman, A. Kasarskis, D. R. Altman, M. Smith, R. Sebra, J. Aberg, F. Krammer, A. García-Sastre, M. Luksza, G. Patel, A. Paniz-Mondolfi, M. Gitman, E. M. Sordillo, V. Simon, H. van Bakel, Introductions and early spread of SARS-CoV-2 in the New York City area. *Science* **369**, 297–301 (2020). doi:10.1126/science.abc1917 Medline
42. S. Riley, H. Wang, O. Eales, C. E. Walters, K. E. C. Ainslie, C. Atchison, C. Fronterre, P. J. Diggle, D. Ashby, C. A. Donnelly, G. Cooke, W. Barclay, H. Ward, A. Darzi, P. Elliott, REACT-1 round 8 interim report: SARS-CoV-2 prevalence during the initial stages of the third national lockdown in England. bioRxiv [Preprint] 22 January 2021. doi:10.1101/2021.01.20.21250158.
43. N. W. Ruktanonchai, J. R. Floyd, S. Lai, C. W. Ruktanonchai, A. Sadilek, P. Rente-Lourenco, X. Ben, A. Carioli, J. Gwinn, J. E. Steele, O. Prosper, A. Schneider, A. Oplinger, P. Eastham, A. J. Tatem, Assessing the impact of coordinated COVID-19 exit strategies across Europe. *Science* **369**, 1465–1470 (2020). doi:10.1126/science.abc5096 Medline
44. P. Lemey, N. Ruktanonchai, S. Hong, V. Colizza, C. Poletto, F. Van den Broeck, M. Gill, X. Ji, A. Levasseur, A. Sadilek, S. Lai, A. Tatem, G. Baele, M. Suchard, S. Dellicour, SARS-CoV-2 European resurgence foretold: interplay of introductions and persistence by leveraging genomic and mobility data. *Nat. Portfolio* [Preprint] 10 February 2021. doi:10.21203/rs.3.rs-208849/v1.
45. E. Volz, V. Hill, J. T. McCrone, A. Price, D. Jorgensen, Á. O'Toole, J. Southgate, R. Johnson, B. Jackson, F. F. Nascimento, S. M. Rey, S. M. Nicholls, R. M. Colquhoun, A. da Silva Filipe, J. Shepherd, D. J. Pascall, R. Shah, N. Jesudason, K. Li, R. Jarrett, N. Pacchiarini, M. Bull, L. Geidelberg, I. Siveroni, I. Goodfellow, N. J. Loman, O. G. Pybus, D. L. Robertson, E. C. Thomson, A. Rambaut, T. R. Connor, C. Koshy, E.

Wise, N. Cortes, J. Lynch, S. Kidd, M. Mori, D. J. Fairley, T. Curran, J. P. McKenna, H. Adams, C. Fraser, T. Golubchik, D. Bonsall, C. Moore, S. L. Caddy, F. A. Khokhar, M. Wantoch, N. Reynolds, B. Warne, J. Maksimovic, K. Spellman, K. McCluggage, M. John, R. Beer, S. Afifi, S. Morgan, A. Marchbank, A. Price, C. Kitchen, H. Gulliver, I. Merrick, J. Southgate, M. Guest, R. Munn, T. Workman, T. R. Connor, COG-UK Consortium, Evaluating the Effects of SARS-CoV-2 Spike Mutation D614G on Transmissibility and Pathogenicity. *Cell* **184**, 64–75.e11 (2021). doi:10.1016/j.cell.2020.11.020 Medline

46. E. B. Hodcroft, M. Zuber, S. Nadeau, K. H. D. Crawford, J. D. Bloom, D. Veesler, T. G. Vaughan, I. Comas, F. G. Candelas, T. Stadler, R. A. Neher, Emergence and spread of a SARS-CoV-2 variant through Europe in the summer of 2020. medRxiv [Preprint] 24 March 2020. doi:10.1101/2020.10.25.20219063.
47. COVID-19 Genomics Consortium UK (CoG-UK), Preliminary genomic characterisation of an emergent SARS-CoV-2 lineage in the UK defined by a novel set of spike mutations (2020); https://virological.org/t/preliminary-genomic-characterisation-of-an-emergent-sars-cov-2-lineage-in-the-uk-defined-by-a-novel-set-of-spike-mutations/563.
48. J. Lu, L. du Plessis, Z. Liu, V. Hill, M. Kang, H. Lin, J. Sun, S. François, M. U. G. Kraemer, N. R. Faria, J. T. McCrone, J. Peng, Q. Xiong, R. Yuan, L. Zeng, P. Zhou, C. Liang, L. Yi, J. Liu, J. Xiao, J. Hu, T. Liu, W. Ma, W. Li, J. Su, H. Zheng, B. Peng, S. Fang, W. Su, K. Li, R. Sun, R. Bai, X. Tang, M. Liang, J. Quick, T. Song, A. Rambaut, N. Loman, J. Raghwani, O. G. Pybus, C. Ke, Genomic Epidemiology of SARS-CoV-2 in Guangdong Province, China. *Cell* **181**, 997–1003.e9 (2020). doi:10.1016/j.cell.2020.04.023 Medline
49. Public Health England, COVID-19: review of disparities in risks and outcomes (2020); https://www.gov.uk/government/publications/covid-19-review-of-disparities-in-risks-and-outcomes.
50. M. U. G. Kraemer, D. A. T. Cummings, S. Funk, R. C. Reiner, N. R. Faria, O. G. Pybus, S. Cauchemez, Reconstruction and prediction of viral disease epidemics. *Epidemiol. Infect.* **147**, 1–7 (2018). Medline
51. N. R. Faria, T. A. Mellan, C. Whittaker, I. M. Claro, D. D. S. Candido, S. Mishra, M. A. E. Crispim, F. C. S. Sales, I. Hawryluk, J. T. McCrone, R. J. G. Hulswit, L. A. M. Franco, M. S. Ramundo, J. G. de Jesus, P. S. Andrade, T. M. Coletti, G. M. Ferreira, C. A. M. Silva, E. R. Manuli, R. H. M. Pereira, P. S. Peixoto, M. U. G. Kraemer, N. Gaburo Jr., C. D. C. Camilo, H. Hoeltgebaum, W. M. Souza, E. C. Rocha, L. M. de Souza, M. C. de Pinho, L. J. T. Araujo, F. S. V. Malta, A. B. de Lima, J. D. P. Silva, D. A. G. Zauli, A. C. S. Ferreira, R. P. Schnekenberg, D. J. Laydon, P. G. T. Walker, H. M. Schlüter, A. L. P. Dos Santos, M. S. Vidal, V. S. Del Caro, R. M. F. Filho, H. M. Dos Santos, R. S. Aguiar, J. L. Proença-Modena, B. Nelson, J. A. Hay, M. Monod, X. Miscouridou, H. Coupland, R. Sonabend, M. Vollmer, A. Gandy, C. A. Prete Jr., V. H. Nascimento, M. A. Suchard, T. A. Bowden, S. L. K. Pond, C.-H. Wu, O. Ratmann, N. M. Ferguson, C. Dye, N. J. Loman, P. Lemey, A. Rambaut, N. A. Fraiji, M. D. P. S. S. Carvalho, O. G. Pybus, S. Flaxman, S. Bhatt, E. C. Sabino, Genomics and epidemiology of the P.1 SARS-CoV-2 lineage in Manaus, Brazil. *Science* **372**, 815–821 (2021). doi:10.1126/science.abh2644 Medline
52. C. M. Saad-Roy, S. E. Morris, C. J. E. Metcalf, M. J. Mina, R. E. Baker, J. Farrar, E. C. Holmes, O. G. Pybus, A. L. Graham, S. A. Levin, B. T. Grenfell, C. E. Wagner, Epidemiological and evolutionary considerations of SARS-CoV-2 vaccine dosing regimes. *Science* **372**, 363–370 (2021). doi:10.1126/science.abg8663 Medline
53. B. T. Grenfell, O. G. Pybus, J. R. Gog, J. L. N. Wood, J. M. Daly, J. A. Mumford, E. C. Holmes, Unifying the epidemiological and evolutionary dynamics of pathogens. *Science* **303**, 327–332 (2004). doi:10.1126/science.1090727 Medline
54. R. N. Thompson, E. M. Hill, J. R. Gog, SARS-CoV-2 incidence and vaccine escape. *Lancet Infect. Dis.* **21**, 913–914 (2021). doi:10.1016/S1473-3099(21)00202-4 Medline
55. B. L. Rice, A. Annapragada, R. E. Baker, M. Bruijning, W. Dotse-Gborgbortsi, K. Mensah, I. F. Miller, N. V. Motaze, A. Raherinandrasana, M. Rajeev, J. Rakotonirina, T. Ramiadantsoa, F. Rasambainarivo, W. Yu, B. T. Grenfell, A. J. Tatem, C. J. E. Metcalf, Variation in SARS-CoV-2 outbreaks across sub-Saharan Africa. *Nat. Med.* **27**, 447–453 (2021). doi:10.1038/s41591-021-01234-8 Medline
56. N. B. Masters, M. C. Eisenberg, P. L. Delamater, M. Kay, M. L. Boulton, J. Zelner, Fine-scale spatial clustering of measles nonvaccination that increases outbreak potential is obscured by aggregated reporting data. *Proc. Natl. Acad. Sci. U.S.A.* **117**, 28506–28514 (2020). doi:10.1073/pnas.2011529117 Medline
57. V. Hill, COG-UK/B.1.1.7_spatial_analysis_UK: V1.0.0. Zenodo (2021); doi:10.5281/zenodo.5085521.
58. O2, Corporate statements; www.o2.co.uk/abouto2/corporate-statements.
59. Newzoo Global Mobile Market Report 2020 (2020); https://newzoo.com/insights/trend-reports/newzoo-global-mobile-market-report-2020-free-version/).
60. M. U. G. Kraemer, A. Sadilek, Q. Zhang, N. A. Marchal, G. Tuli, E. L. Cohn, Y. Hswen, T. A. Perkins, D. L. Smith, R. C. Reiner Jr., J. S. Brownstein, Mapping global variation in human mobility. *Nat. Hum. Behav.* **4**, 800–810 (2020). doi:10.1038/s41562-020-0875-0 Medline
61. Android: market share in the UK 2011-2021; https://www.statista.com/statistics/271240/android-market-share-in-the-united-kingdom-uk/).
62. Coronavirus (COVID-19) Infection Survey: England - office for national statistics; www.ons.gov.uk/peoplepopulationandcommunity/healthandsocialcare/conditionsanddiseases/datasets/coronaviruscovid19infectionsurveydata.
63. X. He, E. H. Y. Lau, P. Wu, X. Deng, J. Wang, X. Hao, Y. C. Lau, J. Y. Wong, Y. Guan, X. Tan, X. Mo, Y. Chen, B. Liao, W. Chen, F. Hu, Q. Zhang, M. Zhong, Y. Wu, L. Zhao, F. Zhang, B. J. Cowling, F. Li, G. M. Leung, Temporal dynamics in viral shedding and transmissibility of COVID-19. *Nat. Med.* **26**, 672–675 (2020). doi:10.1038/s41591-020-0869-5 Medline
64. M. N. Price, P. S. Dehal, A. P. Arkin, FastTree 2—Approximately maximum-likelihood trees for large alignments. *PLOS ONE* **5**, e9490 (2010). doi:10.1371/journal.pone.0009490 Medline
65. M. Hasegawa, H. Kishino, T. Yano, Dating of the human-ape splitting by a molecular clock of mitochondrial DNA. *J. Mol. Evol.* **22**, 160–174 (1985). doi:10.1007/BF02101694 Medline
66. B. Q. Minh, H. A. Schmidt, O. Chernomor, D. Schrempf, M. D. Woodhams, A. von Haeseler, R. Lanfear, IQ-TREE 2: New Models and Efficient Methods for Phylogenetic Inference in the Genomic Era. *Mol. Biol. Evol.* **37**, 1530–1534 (2020). doi:10.1093/molbev/msaa015 Medline
67. P. Sagulenko, V. Puller, R. A. Neher, TreeTime: Maximum-likelihood phylodynamic analysis. *Virus Evol.* **4**, vex042 (2018). doi:10.1093/ve/vex042 Medline
68. V. Hill, G. Baele, Bayesian estimation of past population dynamics in BEAST 1.10 using the Skygrid coalescent model. *Mol. Biol. Evol.* **36**, 2620–2628 (2019). doi:10.1093/molbev/msz172 Medline
69. M. S. Gill, P. Lemey, N. R. Faria, A. Rambaut, B. Shapiro, M. A. Suchard, Improving Bayesian population dynamics inference: A coalescent-based model for multiple loci. *Mol. Biol. Evol.* **30**, 713–724 (2013). doi:10.1093/molbev/mss265 Medline
70. M. A. Suchard, P. Lemey, G. Baele, D. L. Ayres, A. J. Drummond, A. Rambaut, Bayesian phylogenetic and phylodynamic data integration using BEAST 1.10. *Virus Evol.* **4**, vey016 (2018). doi:10.1093/ve/vey016 Medline
71. A. Pope, GB Postcode Area, Sector, District. Edinburgh DataShare (2017); doi:10.7488/ds/1947.
72. B. Ripley, Support Functions and Datasets for Venables and Ripley's MASS [R package MASS version 7.3-53.1] (2021); https://cran.r-project.org/package=MASS.
73. V. Calcagno, Model Selection and Multimodel Inference Made Easy [R package glmulti version 1.0.8] (2020); https://cran.r-project.org/web/packages/glmulti/index.html.

ACKNOWLEDGMENTS

We thank all involved in the collection and processing of SARS-CoV-2 testing and genomic data. We also thank Public Health England (PHE) for making anonymized epidemiological data available for this analysis. We thank the Office of National Statistics (ONS) for their effort to publish the Coronavirus (COVID-19) infection surveys in real-time. **Funding:** VH was supported by the Biotechnology and Biological Sciences Research Council (BBSRC) [grant number BB/M010996/1]. AR acknowledges the support of the Wellcome Trust (Collaborators Award 206298/Z/17/Z – ARTIC network) and the European Research Council (grant agreement no. 725422 – ReservoirDOCS). M.U.G.K. acknowledges support from the Branco Weiss Fellowship. M.U.G.K. and S.D. acknowledge support from the European Union's Horizon 2020 project MOOD (grant agreement no. 874850). O.G.P. and M.U.G.K. acknowledge support from the Oxford Martin School. A.L.B., S.V.S. and M.U.G.K. acknowledge support from the Rockefeller Foundation and Google.org. C.R. was supported by a Fondation Botnar Research Award (Programme grant 6063) and UK Cystic Fibrosis Trust (Innovation Hub Award 001). A.L.B acknowledges support from the Biotechnologyand Biological Sciences Research Council (BBSRC) [grant number BB/M011224/1]. SD acknowledges support from the *Fonds National de la Recherche Scientifique* (FNRS, Belgium). GB acknowledges support from the Research Foundation - Flanders (Fonds voor Wetenschappelijk Onderzoek - Vlaanderen, G0E1420N and G098321N) and from the Interne Fondsen KU Leuven/Internal Funds KU Leuven under grant agreement C14/18/094. COG-UK is supported by funding from the Medical Research Council (MRC) part of UK Research & Innovation (UKRI), the National Institute of Health Research (NIHR) and Genome Research Limited, operating as the Wellcome Sanger Institute. A.OT is supported by the Wellcome Trust Hosts, Pathogens & Global Health Programme [grant number: grant.203783/Z/16/Z] and Fast Grants [award number: 2236]. The contents of this publication are the sole responsibility of the authors and do not necessarily reflect the views of the European Commission or any of the other funders. **Author contributions:** M.U.G.K., A.R., V.H., C.R., S.D., S.V.S, O.G.P. conceived and planned the research. M.U.G.K., V.H., A.R., C.R., S.D., G.B., B.K., A.L.B., S.D., S.G., S.V.S. analyzed the data. M.U.G.K. and O.G.P. wrote the first draft. All authors contributed to writing and interpreting the results. M.U.G.K., A.R., S.V.S., and O.G.P. jointly supervised this work. **Competing interests:** O.G.P., A.R., A.O'T. have undertaken consulting for AstraZeneca relating to the genetic diversity and classification of SARS-CoV-2 lineages. SVS is a paid consultant with Pandefense Advisory and Booz Allen Hamilton; is on the advisory board for BioFire Diagnostics Trend Surveillance, which includes paid consulting; and holds unexercised options in Iliad Biotechnologies. These entities provided no financial support associated with this research, did not have a role in the design of this study, and did not have any role during its execution, analyses, interpretation of the data and/or decision to submit. **Data and materials availability:** Aggregated epidemiological data used in this study are available from https://coronavirus.data.gov.uk/details/download. SARS-CoV-2 infection survey data are available via the Office of National Statistics (ONS) and available from https://www.ons.gov.uk/peoplepopulationandcommunity/healthandsocialcare/conditionsanddiseases/datasets/coronaviruscovid19infectionsurveydata. Raw epidemiological SARS-CoV-2 line list data are available via Public Health England (PHE) and aggregated statistics are available via Github. All genomes, phylogenetic trees, basic metadata are available from the COG-UK consortium website (https://www.cogconsortium.uk/data). The O2 aggregated, anonymised mobile data insights dataset is not publicly available owing to stringent licensing agreements. Information on the process of requesting access to the O2 aggregated mobile data insights dataset is available o2@businesso2.co.uk. The Google COVID-19 Aggregated Mobility Research Dataset is not publicly available owing to stringent licensing agreements. Information on the process of requesting access to the Google mobility data are available from sadilekadam@google.com. Code and data are available on the following GitHub repository https://github.com/COG-UK/B.1.1.7_spatial_analysis_UK and permanently on Zeonodo (*57*). This work is licensed under a Creative Commons Attribution 4.0 International (CC BY 4.0) license, which permits unrestricted use, distribution, and reproduction in any medium, provided the original work is properly cited. To view a copy of this license, visit https://creativecommons.org/licenses/by/4.0/. This license does not apply to figures/photos/artwork or other content included in the article that is credited to a third party; obtain authorization from the rights holder before using such material.

SUPPLEMENTARY MATERIALS

science.sciencemag.org/cgi/content/full/science.abj0113/DC1
Materials and Methods
Figs. S1 to S19
Tables S1 to S3
COVID-19 Genomics UK (CoG-UK) Consortium Author List
References (*58*–*73*)
MDAR Reproducibility Checklist

16 April 2021; accepted 12 July 2021
Published online 22 July 2021
10.1126/science.abj0113

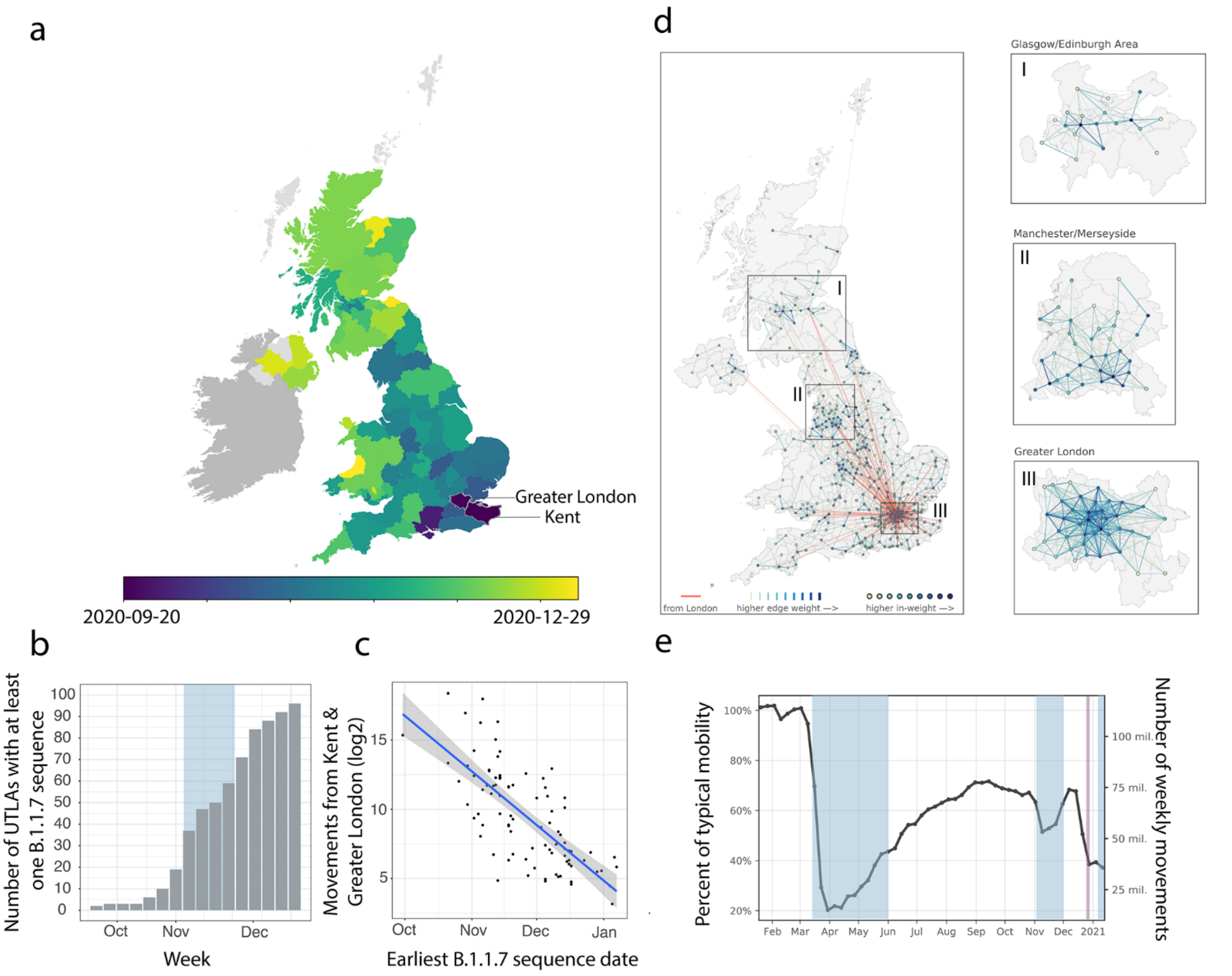

Fig. 1. Human mobility and spatial expansion of B.1.1.7 across the UK. (**A**) Map at the UTLA level (*n* = 115) of arrival dates of lineage B.1.1.7. Darker colors indicate earlier dates and lighter colors later dates. Arrival time is defined as the earliest sampling date of a B.1.1.7 genomic sequence in each UTLA. (**B**) Cumulative number of UTLAs in which B.1.1.7 has been detected, in 7-day intervals. The blue shaded area indicates the period of the second lockdown in England. (**C**) Relationship between the arrival time of B.1.1.7 and estimated number of movements from Kent and London during February 2020 for each UTLA (Pearson's $r = -0.73$, 95% CI: −0.61; −0.81, P value < 0.001, see materials an methods). (**D**) Human mobility at the UK Local Authority District Level (LAD) (table S2) during the epidemiological week 29 November–5 December 2020. Thicker lines (edges) represent more movements between regions. Nodes with larger absolute incoming movements are shown with darker colors. Red lines show movements from Greater London. Insets show mobility within three UK metropolitan areas. (**E**) Trends in human mobility across the UK (representing movements between but not within LADs). The blue shaded areas indicate the period of the first, second and third lockdown in England. Dark red shows the timing (20 December 2020) of the Tier 4 restrictions imposed in southeast England, including London (*23*).

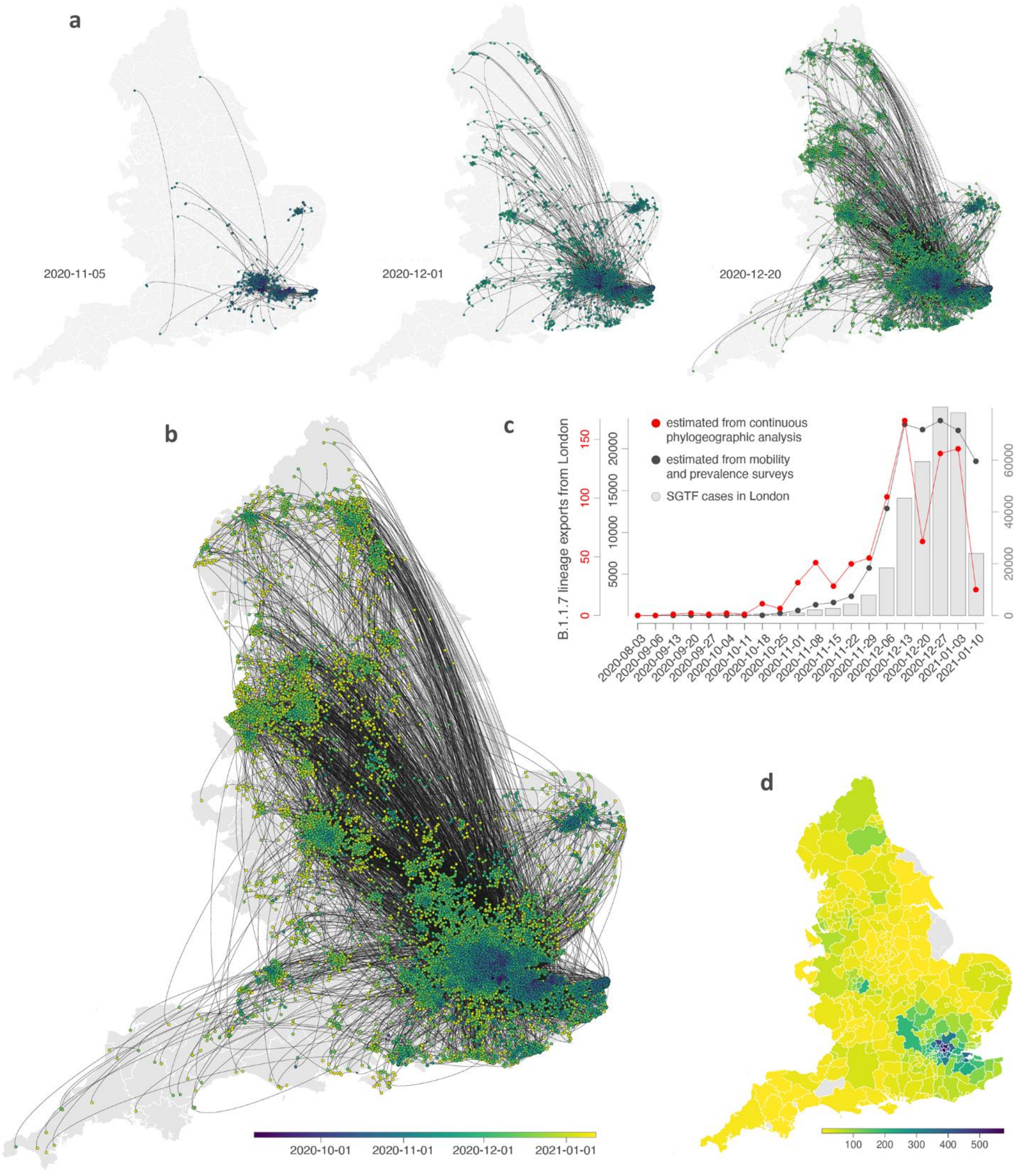

Fig. 2. Spatial emergence dynamics of SARS-CoV-2 lineage B.1.1.7 in England. (A and B) Continuous phylogeographic reconstruction with phylogeny nodes colored according to their time of occurrence and dispersal direction of phylogeny branches indicated by edge curvature (anti-clockwise). From left-to-right, data to 5 November, 1 December and 20 December 2020, respectively. (B) Map of the entire reconstruction, up to 19 January 2021. (C) Estimated number of weekly exports of lineage B.1.1.7 from the Greater London area, inferred from the continuous phylogeographic analysis (red), and estimated from mobility and prevalence survey data (black). (D) Estimated number of cumulative B.1.1.7 introductions inferred from phylogeographic analysis into each administrative area (UTLA) by 12 December 2020.

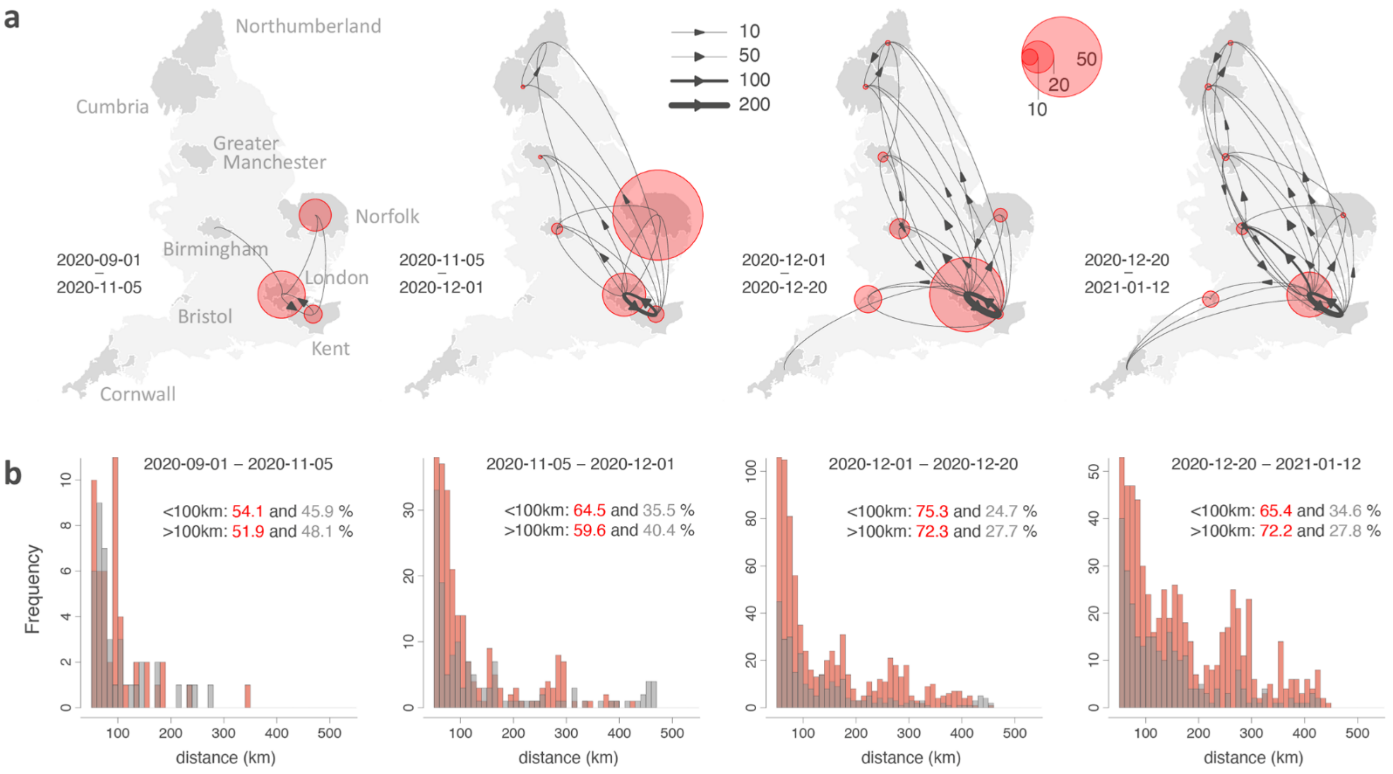

Fig. 3. Spatial structure of B.1.1.7 lineage dispersal in England from phylogeographic reconstruction. (**A**) Curved arrows and line thicknesses show the direction and intensity of B.1.1.7 lineage flows among regions. Red circles indicate, for a given location, the ratio of inferred local movements to inferred importations into that location. Four time periods are shown (left to right) and roughly correspond to (i) pre second lockdown, (ii) second lockdown, (iii) post second lockdown and (iv) implementation of Tier 4 restrictions in southeast England. (**B**) Distribution of the geographic distances of phylogenetic lineage movement events (>50 km); those from Greater London are in red and those from other locations are in grey.

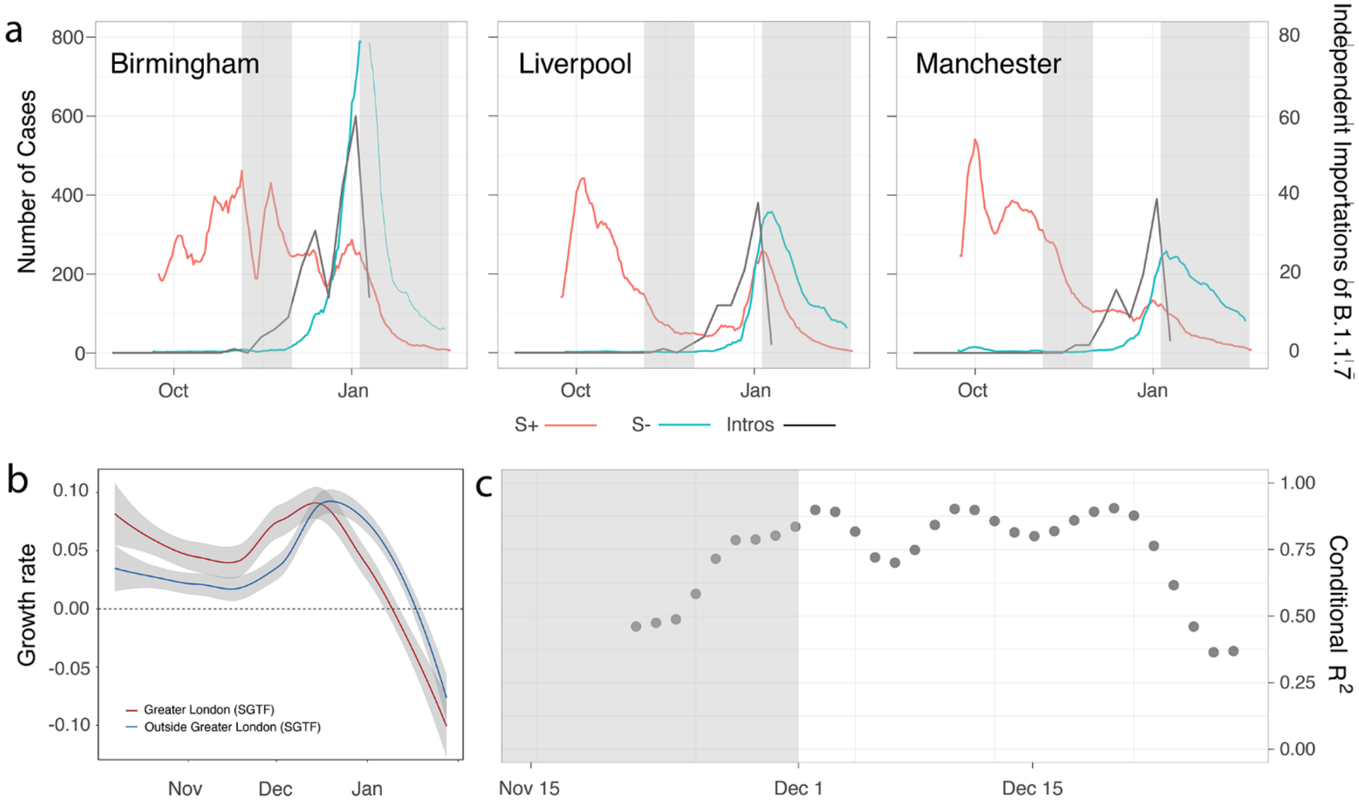

Fig. 4. Case growth rates of B.1.1.7 are correlated with human mobility and attack rates across the United Kingdom. (**A**) 7-day rolling average number of cases reported that had the S-gene target failure (SGTF) in green and cases reported with non-SGTF (red) for three selected Lower Tier Local Authorities [LTLAs (table S2) Birmingham, Liverpool and Manchester]. The black line shows the weekly number of independent introductions estimated from the phylogeographic analysis. Grey shaded area indicates the timing of the second (5 November - 1 December) and start of the third (5 January) English lockdown. (**B**) Rolling 7-day average growth rates of SGTF cases in Greater London (red line) and outside of Greater London (blue line). (**C**) Association between per-region (LTLA) difference between SGTF and non-SGTF case growth rates (corrected to account for differences through time in sampling intensity of SGTF cases) and number of B.1.1.7 importations into that region, as estimated from prevalence surveys and human mobility (grey dots; Fig. 2C) (materials and methods). Grey area shows the time of the second English lockdown. Modelling results for SGTF growth rates are shown in fig. S9 and regression results under different assumptions about the frequency of SGTF are shown in fig. S10.

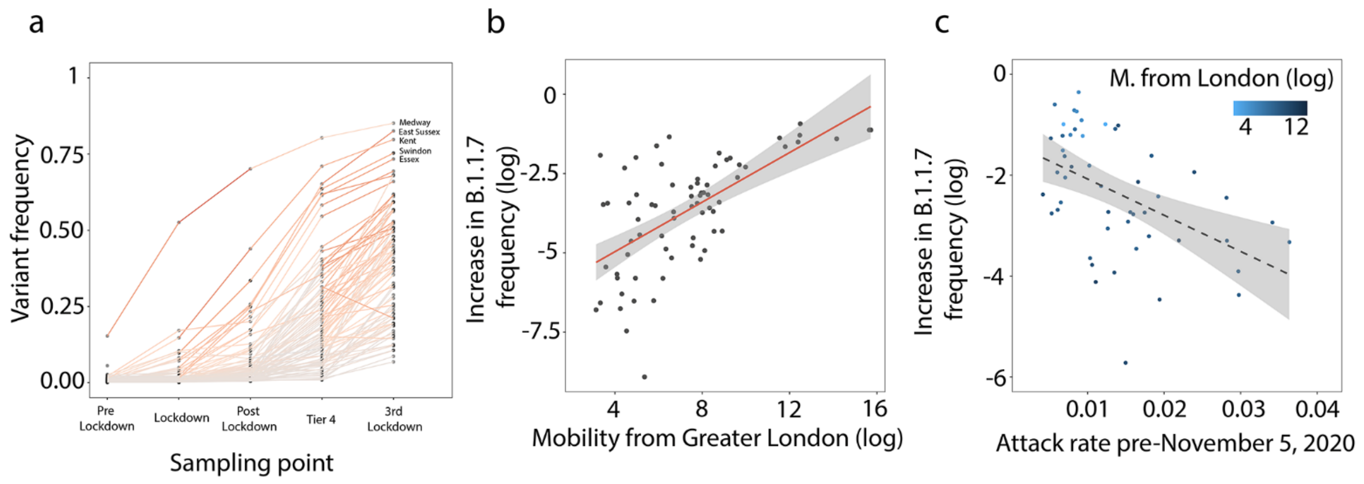

Fig. 5. (A) Frequency of B.1.1.7 at the UTLA level at different sampling times (pre-lockdown refers to dates prior to 5 November; lockdown 5–30 November; post-lockdown 1–15 December; Tier 4, 16–31 December; and the most recent sampling point 1–12 January, Materials and Methods). (B) Increase in the frequency of B.1.1.7 sampled genomes between 2 - 16 December 2020 is associated with mobility from Greater London. (C) Increase in the frequency of B.1.1.7 sampled genomes at the UTLA level is associated with previous attack rates in each location. Results for equivalent analyses of SGTF data are similar and shown in supplementary materials.
